# Supplementary material for: ACKR3 regulates platelet activation and ischemia-reperfusion tissue injury
Source: Nat Commun. 2022 Apr 5;13:1823. doi: 10.1038/s41467-022-29341-1 (PMC8983782; doi:10.1038/s41467-022-29341-1)
Supplement: Supplementary file 1 — Supplementary Information [file 41467_2022_29341_MOESM1_ESM.pdf]

Supplementary Table 1: Major Resource Table

Animals (in vivo studies)

Species	Vendor or Source	Background Strain	Sex	Persistent ID/ URL
**C57BL/6J**	The Jackson Laboratories, Bar Harbor, Maine, USA	-	♂/♀	https://www.jax.org/strain/000664
**C57BL/6-Tg(Pf4-cre)Q3Rsko/J**	The Jackson Laboratories, Bar Harbor, Maine, USA	C57BL/6J	♂/♀	https://www.jax.org/strain/008535
**B6.Cg-Ackr3^{tm1Fma}/J**	Dr. Fabienne Mackay, Victor Chang Cardiac Research Institute, Darlinghurst, Australia	C57BL/6J	♂/♀	https://www.jax.org/strain/033828
**B6.129(Cg)-Gt(ROSA)26Sortm4(ACTB-tdTomato,-EGFP)Luo/J**	The Jackson Laboratories, Bar Harbor, Maine, USA	C57BL/6J	♂/♀	https://www.jax.org/strain/007676

Genetically Modified Animals

	Species	Vendor or Source	Background Strain	Persistent ID/ URL
**B6.Cg-Thy1a-(Ackr3)Ackr3^{tm1Fma}-Tg(Pf4-icre)Q3Rsko/J**				
**Parent - male**	**C57BL/6-Tg(Pf4-cre)Q3Rsko/J**	The Jackson Laboratories, Bar Harbor, Maine, USA	C57BL/6J	https://www.jax.org/strain/008535
**Parent - female**	**B6.Cg-Ackr3^{tm1Fma}/J**	Dr. Fabienne Mackay, Victor Chang Cardiac Research Institute, Darlinghurst, Australia	C57BL/6J	https://www.jax.org/strain/033828
**B6.Cg-Thy1a-(Ackr3)Ackr3^{tm1Fma}-Tg(Pf4-cre)Q3Rsko-Tg(ROSA$^{mT/mG}$)/J**				
**Parent - male**	B6.Cg-Thy1a-(Ackr3)Ackr3^{tm1Fma}-Tg(Pf4-icre)Q3Rsko/J	breed in house	C57BL/6J	-
**Parent - female**	B6.129(Cg)-Gt(ROSA)26Sortm4(ACTB-tdTomato,-EGFP)Luo/J	Laboratories, Bar Harbor, Maine, USA	C57BL/6J	https://www.jax.org/strain/007676

Antibodies

antigen	clone	company	order number	Dilution
**Immunohistochemistry**				
anti-CD45R/B220	RA3-6B2	BD Pharmingen, Franklin Lakes, New Jersey, USA	553084	1:3000
anti-CD3	SP7	Zytomed Systems GmbH, Berlin, Germany	RBK024	1:500
anti-CD42b	SP219	Abcam, Cambrigde, UK	ab183345	1:200
anti-Ly6G	1A8	BD Pharmingen, Franklin Lakes, New Jersey, USA	551459	1:800
anti-MHCII	M5/114.15.2	Novus Biologicals Littelton CO, USA	NBP1-43312	1:500
AffinitiyPure rabbit anti-rat IgG (h+L)	-	Jackson Immunoreserach Europe Ltd. Cambridgeshire, UK	312-005-045	1:250
AffinityPure goat anti-rabbit IgG (H+L)	-	Jackson Immunoreserach Europe Ltd. Cambridgeshire, UK	111-035-144	1:100
anti-TNFalpha	-	Novus Biologicals Littelton CO, USA	NBP1-19532	1:500
anti-IL-1 beta/IL-1F2	-	Novus Biologicals Littelton CO, USA	NB600-633	1:200
anti-CD31	-	Abcam, Cambrigde, UK	ab124432	1:50
**Immunofluorescence**				
anti-CD42b	SP219	Abcam, Cambrigde, UK	ab183345	1:100
anti-rabbit IgG-AlexaFluor488 (H+L)	-	Invitrogen, Karlsbad, California, USA	A22106	1:200
anti-CD62P	-	Emfret Analytics GmbH & Co. Kg, Eibelstadt, Germany	M130-0	1:100
anti-CXCR7	-	Abcam, Cambrigde, UK	Ab72100	1:100
anti-NeuN	-	Merck, Darmstadt, Germany	ABN91	1:1000
anti-GFPA	-	Thermofisher, Waltham, Massachusetts, USA	13-0300	1:200
anti-Iba1	-	Abcam, Cambrigde, UK	ab5076	1:100
anti-Ly6G	-	Biolegend, San Diego, California, USA	127601	1:100
anti-CD3	-	BioRad, Hercules, Kalifornien, USA	MCA1477	1:100
anti-rabbit IgG-AlexaFluor568 (H+L)	-	Invitrogen, Karlsbad, California, USA	A11061	1:200
Rabbit IgG Control	-	R&D Systems, Minneapolis, Minnesota, USA	AB-105-C	1:100
Phalloidin488	-	Invitrogen, Karlsbad, California, USA	A12379	1:200
Rhodamine Phalloidin	-	Invitrogen, Karlsbad, California, USA	R415	1:200
anti-rat IgG-AlexaFluor647 (H+L)	-	Invitrogen, Karlsbad, California, USA	A21247	1:500
anti-goat IgG-AlexaFluor555 (H+L)	-	Invitrogen, Karlsbad, California, USA	A21432	1:500
anti-rat IgG-AlexaFluor555 (H+L)	-	Invitrogen, Karlsbad, California, USA	A21434	1:500
anti-chicken IgY-AlexaFluor488 (H+L)	-	Jackson ImmunoResearch Europe Ltd, London, UK	703-545-155	1:500
**Immunoblot**				

anti-CXCR7	-	Novus Biologicals Littelton CO, USA	NBP1-31309	1:1000
anti-CXCR7	-	Abcam, Cambrigde, UK	Ab72100	1:200
anti-mouse alpha-Tubulin	DM1A	Cell Signaling Technology, Denvers Massachusetts, USA	3873	1:1000
IRDye 680LT goat anti-rabbit IgG	-	Li-Cor Biosciences GmbH, Nabraska, USA	P/N 925-68021	1:15000
IRDye 800CW goat anti-mouse IgG	-	Li-Cor Biosciences GmbH, Nabraska, USA	P/N 925-32210	1:15000
**Flow Cytometry**				
anti-CD62P	CLB-Thromb/6	Beckman Coulter, Brea, California, USA	A07790	1:10
Mouse monoclonal anti-human CXCR7-PE	358426	R&D Systems, Minneapolis, Minnesota, USA	FAB42271P	1:100
Mouse IgG2b-PE	20102	R&D Systems, Minneapolis, Minnesota, USA	IC003P	1:100
Annexin V FITC		ImmunoTools, Friesoythe, Germany	31490013	1:50
Anti-human GPIb-FITC	SZ2	Beckman Coulter, Brea, California, USA	IM0648	1:50
rat anti-mouse CD42b/GPIbα-FITC	Xia.G7	Emfret Analytics GmbH & Co. Kg, Eibelstadt, Germany	M042-1	1:10
rat anti mouse CD42b/GPIbα-Dylight 648	Xia.G5	Emfret Analytics GmbH & Co. Kg, Eibelstadt, Germany	M040-3	1:10
rat anti-mouse CD62P-FITC	Wug.E9	Emfret Analytics GmbH & Co. Kg, Eibelstadt, Germany	M130-1	1:10
rat-anti mouse CD45R-BUV395	RA3-6B2	BD Biosciences, San Jose, California, USA	563793	1:400
rat-anti mouse I-A/I-E-BUV496	M5/114.15.2	BD Biosciences, San Jose, California, USA	750281	1:250
rat-anti mouse CD8a-BUV737	53-6.7	BD Biosciences, San Jose, California, USA	612759	1:1600
mouse-anti mouse XCR1-BV421	ZET	Biolegend, San Diego, California, USA	148216	1:1280
rat-anti mouse Ly6C-PacificBlue	HK1.4	Biolegend, San Diego, California, USA	128014	1:2000
rat-anti mouse CD4-BV510	GK1.5	Biolegend, San Diego, California, USA	100449	1:320
mouse-anti mouse NK1.1-BV650	PK136	Biolegend, San Diego, California, USA	108736	1:640
rat-anti mouse Ly6G-BV711	1A8	Biolegend, San Diego, California, USA	127643	1:640
hamster-anti mouse CD11c-BV785	N418	Biolegend, San Diego, California, USA	117335	1:160
hamster-anti mouse CD3e-FITC	145-2C11	Miltenyi, Bergisch-Gladbach, Germany	130-092-962	1:40
rat-anti mouse CD45-PerCp	30-F11	Biolegend, San Diego, California, USA	103130	1:320
mouse-anti mouse CD64-PE	X54-5/7.1	Biolegend, San Diego, California, USA	139304	1:320
rat-anti mouse CD25-PE-Cy7	PC61	Biolegend, San Diego, California, USA	102016	1:320
rat-anti mouse CD172a-APC	P84	Biolegend, San Diego, California, USA	144014	1:400
rat-anti mouse/human CD11b-AlexaFluor700	M1/70	Biolegend, San Diego, California, USA	101222	1:2000
rat-anti mouse CD19-APC-Cy7	6D5	Biolegend, San Diego, California, USA	115530	1:800
anti-CCR2-PE	475301	R&D Systems, Minneapolis, Minnesota, USA	FAB5538P	1:25
anti-CD11b PerCP-CY5.5/APC-CY7	M1/70	Biolegend, San Diego, California, USA	10226	1:100

anti-CD11c-PerCP-Cy5.5	N418	Biolegend, San Diego, California, USA	N418	1:50
anti-CD45-BV421	30-F11	Biolegend, San Diego, California, USA	103134	1:100
CD64-PECy7	X54-5/7.1	Biolegend, San Diego, California, USA	X54-5/7.1	1.100
IA/IE-PE A488	M5/114.15.2	Biolegend, San Diego, California, USA	107616	1:100
anti-IL-1b PE	166931	R&D Systems, Minneapolis, Minnesota, USA	IC4013P	1:50
anti-Ly6C APC	HK1.4	Biolegend, San Diego, California, USA	128010	1:100
anti-Ly6G A700	1A8	Biolegend, San Diego, California, USA	127622	1:200
anti-TNF APC-Cy7	MP6-XT22	BD Biosciences, San Jose, California, USA	560658	1:50
**Intravital Microscopy**				
anti-GPIbβ-FITC	-	Emfret Analytics GmbH & Co. Kg, Eibelstadt, Germany	X488	0.1 µg Ab / 1 g body weight

Reagents and Chemicals

**reagent**	**company**	**order number**
$DiOC_6$	Sigma Aldrich Co. St. Luis, Missouri, USA	318426
fura-2 acetoxymethylester	Invitrogen, Carlsbad, California, USA	F1221
DAPI	Invitrogen, Karlsbad, California, USA	D1306
pluronic F-127	Biotium, Hayward, California, USA	59000
recombinant human CCl2/MCP1	R&D Systems, Minneapolis, Minnesota, USA	279-MC-010
Apyrase from potatoes	Sigma Aldrich Co. St. Luis, Missouri, USA	A6535
CRP-XL	CambCol Laboratories, Cambridge, UK	-
ABT-737	Selleck Chemicals GmbH, Houston, Texas, USA	S1002
Prostaglandin I2	Merck, Darmstadt, Germany	P6188
Thrombin from human Plasma	F. Hoffmann La-Roche AG, Basel, Switzerland	10602400001
Fibrinogen from human plasma	Sigma Aldrich Co. St. Luis, Missouri, USA	F3879
Collagen reagent HORM	Takeda, Linz, Austria	-
Ficoll-plaque	Merck, Darmstadt, Germany	GE-17-1440-03
RMPI-1640	Gibco, Carlsbad, California, United States	R8758
DMEM	Gibco, Carlsbad, California, United States	41995-039
10% foetal calf serum	Fisher Scientific, Waltham, Massachusetts, USA	11580516
1 % Penicillin/Streptomycin	Sigma Aldrich Co. St. Luis, Missouri, USA	P4333
TPO	ImmunoTools, Friesoythe, Germany	12343615
Bovine serum albumin	Applichem, Darmstadt, Germany	A1391
DMSO	Applichem, Darmstadt, Germany	A3672

Evans blue dye	Sigma Aldrich Co. St. Luis, Missouri, USA	E2129
TTC dye	Sigma Aldrich Co. St. Luis, Missouri, USA	T8877
Dubecco’s phosphate buffered saline	Sigma Aldrich Co. St. Luis, Missouri, USA	D8537
Fentadon (Fentanyl)	Dechra veterinary products, Aulendorf, Germany	-
Sedator (Medetomidin)	Dechra veterinary products, Aulendorf, Germany	-
Midazolam-hameln	Hameln Pharmaceuticals GmbH, Hameln, Germany	-
Buprenovet sine	Elanco, Greenfield, Indiana, USA	-
LIVE/DEAD Fixable Blue Dead Cell Stain Kit	Fisher Scientific, Waltham, Massachusetts USA; L34962; 1:1000	

ACKR3 agonist and controls

reagent	company	order number		
ACKR3 Agonist VUF22107	Merck, Darmstadt, Germany	239824	$C_{27}H_{35}FN_2O_4$	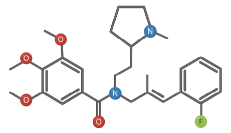
ACKR3 Agonist C10	Enamine Ltd., Kyiv, Ukraine	Z645919236	$C_{16}H_{20}N_6OS$	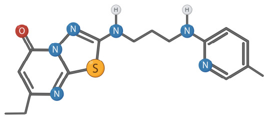
C46 Control	Enamine Ltd., Kyiv, Ukraine	Z373658330	$C_{15}H_{15}N_3O_4$	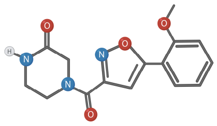

Primer

primer	sequence
mACKR3-Forward	5‘-TCCAGGTTGGCAGATGGATATTG-3‘
mACKR3-Reverse	5’-TGAAATCAGCATGATACAGGGTCC-3’
Pf4-Cre-Forward_1	5’-CCCATACAGCACACCTTTTG-3’
Pf4-Cre-Reverse_1	5’-TGCACAGTCAGCAGGTT-3’
Pf4-Cre-Forward_2	5’-CAAATGTTGCTTGTCTGGTG-3’
Pf4-Cre-Reverse_2	5’-GTCAGTCGAGTGCACAGTTT-3’

Supplementary Table 2:
Baseline characteristics of CAD patients stratified according to CCS and ACS. Continuous variables were compared using unpaired t-tests (two-sided p-values). Dichotomous variables were compared using cross-tabulations with chi-square tests (two-sided p-values).

	**All n=389**	**CCS n=184**	**ACS n=205**	**p-value**
Age (mean ± SD)	68.2 (±12.1)	69.1 (±10.6)	67.4 (±13.2)	0.139
Gender (male)	277 (71.2%)	132 (71.7%)	145 (70.7%)	0.819
LVEF% at admission (mean ± SD)	50.3 (±11.0)	52.3 (±10.4)	47.8 (±11.0)	**<0.001**
**Cardiovascular risk factors (%)**				
Arterial hypertension	338 (86.9%)	170 (92.4%)	168 (82.0%)	**0.003**
Hyperlipidaemia	230 (59.1%)	126 (68.5%)	104 (50.7%)	**<0.001**
Diabetes mellitus type 2	120 (30.8%)	65 (35.3%)	55 (26.8%)	0.083
Smoking	128 (32.9%)	54 (29.3%)	74 (36.1%)	0.130
**Medication on admission (%)**				
ASA	205 (52.7%)	122 (66.3%)	83 (40.5%)	**<0.001**
Clopidogrel	55 (14.1%)	36 (19.6%)	19 (9.3%)	**0.004**
Prasugrel	17 (4.4%)	10 (5.4%)	7 (3.4%)	0.342
Ticagrelor	24 (6.2%)	13 (7.1%)	11 (5.4%)	0.504
ACE inhibitors	168 (43.2%)	92 (50.0%)	76 (37.1%)	**0.010**
ARBs	70 (18.0%)	43 (23.4%)	27 (13.2%)	**0.009**
Beta blockers	220 (56.6%)	136 (73.9%)	84 (41.0%)	**<0.001**
Statins	190 (48.8%)	111 (60.3%)	79 (38.5%)	**<0.001**
**Plasma lipid profile (mean ± SD)**				
Total cholesterol (mg/dl)	172.0 (±43.5)	168.7 (±37.5)	177.5 (±51.7)	0.168
LDL cholesterol (mg/dl)	95.4 (±12.2)	96.3 (±42.9)	93.5 (±39.9)	0.746
HDL cholesterol (mg/dl)	46.0 (±12.2)	47.4 (±13.2)	43.0 (±9.1)	**0.049**
Triglycerides (mg/dl)	152.5 (±78.0)	163.4 (±80.8)	129.7 (±67.3)	**0.035**

Supplementary Table 3: NanoString mRNA profiling. Complete list of the 254 genes tested with name and accession number in alphabetical order.

Gene name	Accession #
**Ager**	NM_007425.2
**Alox12**	NM_007440.4
**Alox15**	NM_009660.3
**Alox5**	NM_009662.2
**Areg**	NM_009704.3
**Arg1**	NM_007482.3
**Atf2**	NM_001025093.1
**Bcl2l1**	NM_009743.4
**Bcl6**	NM_009744.3
**Birc2**	NM_007465.2
**C1qa**	NM_007572.2
**C1qb**	NM_009777.2
**C1ra**	NM_023143.3
**C1s**	NM_144938.2
**C2**	NM_013484.2
**C3**	NM_009778.2
**C3ar1**	NM_009779.2
**C4a**	NM_011413.2
**C6**	NM_016704.2
**C7**	XM_356827.6
**C8a**	NM_146148.1
**C8b**	NM_133882.2
**C9**	NM_013485.1
**Ccl11**	NM_011330.3
**Ccl17**	NM_011332.2
**Ccl19**	NM_011888.2
**Ccl2**	NM_011333.3
**Ccl20**	NM_016960.1
**Ccl21a**	NM_011124.4
**Ccl22**	NM_009137.2

Gene name	Accession #
**Ccl24**	NM_019577.4
**Ccl3**	NM_011337.1
**Ccl4**	NM_013652.1
**Ccl5**	NM_013653.1
**Ccl7**	NM_013654.2
**Ccl8**	NM_021443.2
**Ccr1**	NM_009912.4
**Ccr2**	NM_009915.2
**Ccr3**	NM_009914.4
**Ccr4**	NM_009916.2
**Ccr7**	NM_007719.2
**Cd163**	NM_053094.2
**Cd4**	NM_013488.2
**Cd40**	NM_011611.2
**Cd40lg**	NM_011616.2
**Cd55**	NM_010016.2
**Cd86**	NM_019388.3
**Cdc42**	NM_009861.1
**Cebpb**	NM_009883.3
**Cfb**	NM_008198.2
**Cfd**	NM_013459.1
**Cfl1**	NM_007687.5
**Chi3l3**	NM_009892.1
**Creb1**	NM_133828.2
**Crp**	NM_007768.4
**Csf1**	NM_001113530.1
**Csf2**	NM_009969.4
**Csf3**	NM_009971.1
**Cxcl1**	NM_008176.1
**Cxcl10**	NM_021274.1

Gene name	Accession #
**Cxcl2**	NM_009140.2
**Cxcl3**	NM_203320.2
**Cxcl5**	NM_009141.2
**Cxcl9**	NM_008599.2
**Cxcr1**	NM_178241.4
**Cxcr2**	NM_009909.3
**Cxcr4**	NM_009911.3
**Cysltr1**	NM_021476.4
**Cysltr2**	NM_001162412.1
**Daxx**	NM_007829.3
**Ddit3**	NM_007837.3
**Defa-rs1**	NM_007844.2
**Elk1**	NM_007922.4
**Fasl**	NM_010177.3
**Flt1**	NM_010228.3
**Fos**	NM_010234.2
**Fxyd2**	NM_052823.2
**Gnaq**	NM_008139.5
**Gnas**	NM_010309.3
**Gnb1**	NM_008142.3
**Gngt1**	NM_010314.2
**Gpr44**	NM_009962.2
**Grb2**	NM_008163.3
**H2-Ea-ps**	NM_010381.2
**H2-Eb1**	NM_010382.2
**Hc**	NM_010406.1
**Hdac4**	NM_207225.1
**Hif1a**	NM_010431.2
**Hmgb1**	NM_010439.3
**Hmgb2**	NM_008252.3

Gene name	Accession #
**Hmgn1**	NM_008251.3
**Hras1**	NM_008284.2
**Hsh2d**	NM_197944.1
**Hspb1**	NM_013560.2
**Hspb2**	NM_024441.3
**Ifi27l2a**	NM_029803.1
**Ifi44**	NM_133871.2
**Ifit1**	NM_008331.2
**Ifit2**	NM_008332.2
**Ifit3**	NM_010501.1
**Ifna1**	NM_010502.2
**Ifnb1**	NM_010510.1
**Ifng**	NM_008337.1
**Iigp1**	NM_021792.3
**Il10**	NM_010548.1
**Il10rb**	NM_008349.5
**Il11**	NM_008350.2
**Il12a**	NM_008351.1
**Il12b**	NM_008352.1
**Il13**	NM_008355.2
**Il15**	NM_008357.1
**Il17a**	NM_010552.3
**Il18**	NM_008360.1
**Il18rap**	NM_010553.2
**Il1a**	NM_010554.4
**Il1b**	NM_008361.3
**Il1r1**	NM_001123382.1
**Il1rap**	NM_008364.2
**Il1rn**	NM_031167.4
**Il2**	NM_008366.3

Gene name	Accession #
**Il21**	NM_021782.2
**Il22**	NM_016971.1
**Il22ra2**	NM_178258.5
**Il23a**	NM_031252.1
**Il23r**	NM_144548.1
**Il3**	NM_010556.4
**Il4**	NM_021283.1
**Il5**	NM_010558.1
**Il6**	NM_031168.1
**Il6ra**	NM_010559.2
**Il7**	NM_008371.2
**Il9**	NM_008373.1
**Irf1**	NM_008390.1
**Irf3**	NM_016849.3
**Irf5**	NM_012057.3
**Irf7**	NM_016850.2
**Itgb2**	NM_008404.4
**Jun**	NM_010591.2
**Keap1**	NM_016679.4
**Kng1**	NM_023125.3
**Limk1**	NM_010717.2
**Lta**	NM_010735.1
**Ltb**	NM_008518.2
**Ltb4r1**	NM_008519.2
**Ltb4r2**	NM_020490.2
**Ly96**	NM_016923.1
**Maff**	NM_010755.3
**Mafg**	XM_001002362.1
**Mafk**	NM_010757.2
**Map2k1**	NM_008927.3

Gene name	Accession #
**Map2k4**	NM_009157.4
**Map2k6**	NM_011943.2
**Map3k1**	NM_011945.2
**Map3k5**	NM_008580.4
**Map3k7**	NM_172688.2
**Map3k9**	NM_177395.4
**Mapk1**	NM_001038663.1
**Mapk14**	NM_011951.2
**Mapk3**	NM_011952.2
**Mapk8**	NM_016700.3
**Mapkapk2**	NM_008551.1
**Mapkapk5**	XM_990515.1
**Masp1**	NM_008555.2
**Masp2**	NM_010767.3
**Max**	NM_008558.1
**Mbl2**	NM_010776.1
**Mef2a**	XM_976032.1
**Mef2b**	NM_001045484.1
**Mef2c_Mm**	NM_025282.2
**Mef2d**	NM_133665.3
**Mknk1**	NM_021461.4
**Mmp3**	NM_010809.1
**Mmp9**	NM_013599.2
**Mrc1**	NM_008625.1
**Mx1**	NM_010846.1
**Mx2**	NM_013606.1
**Myc**	NM_010849.4
**Myd88**	NM_010851.2
**Myl2**	NM_010861.3
**Nfatc3**	NM_010901.2

Gene name	Accession #
**Nfe2l2**	NM_010902.3
**Nfkb1**	NM_008689.2
**Nlrp3**	NM_145827.3
**Nod1**	NM_172729.2
**Nod2**	NM_145857.2
**Nos2**	NM_010927.3
**Nox1**	NM_172203.1
**Nr3c1**	NM_008173.3
**Oas1a**	NM_145211.2
**Oas2**	NM_145227.2
**Oasl1**	NM_145209.2
**Pdgfa**	NM_008808.3
**Pik3c2g**	NM_011084.2
**Pla2g4a**	NM_008869.2
**Plcb1**	NM_019677.1
**Ppp1r12b**	NM_001081307.1
**Prkca**	NM_011101.3
**Prkcb**	NM_008855.2
**Ptger1**	NM_013641.2
**Ptger2**	NM_008964.4
**Ptger3**	NM_011196.2
**Ptger4**	NM_008965.1
**Ptgfr**	NM_008966.3
**Ptgir**	NM_008967.3
**Ptgs1**	NM_008969.3
**Ptgs2**	NM_011198.3
**Ptk2**	NM_007982.2
**Rac1**	NM_009007.2
**Raf1**	NM_029780.3
**Rapgef2**	NM_001099624.2

Gene name	Accession #
**Rela**	NM_009045.4
**Relb**	NM_009046.2
**Retnla**	NM_020509.3
**Rhoa**	NM_016802.4
**Ripk1**	NM_009068.3
**Ripk2**	NM_138952.3
**Rock2**	NM_009072.2
**Rps6ka5**	NM_153587.2
**Shc1**	NM_011368.4
**Smad7**	NM_001042660.1
**Stat1**	NM_009283.3
**Stat2**	NM_019963.1
**Stat3**	NM_213659.2
**Tbxa2r**	NM_001277265.1
**Tcf4**	NM_013685.1
**Tgfb1**	NM_011577.1
**Tgfb2**	NM_009367.1
**Tgfb3**	NM_009368.2
**Tgfbr1**	NM_009370.2
**Tlr1**	NM_030682.1
**Tlr2**	NM_011905.2
**Tlr3**	NM_126166.2
**Tlr4**	NM_021297.2
**Tlr5**	NM_016928.2
**Tlr6**	NM_011604.3
**Tlr7**	NM_133211.3
**Tlr8**	NM_133212.2
**Tlr9**	NM_031178.2
**Tnf**	NM_013693.1
**Tnfaip3**	NM_009397.2

Gene name	Accession #
**Tnfsf14**	NM_019418.2
**Tollip**	NM_023764.3
**Tradd**	NM_001033161.2
**Traf2**	NM_009422.2
**Trem2**	NM_031254.2
**Tslp**	NM_021367.1
**Twist2**	NM_007855.2
**Tyrobp**	NM_011662.2
**Cltc**	NM_001003908.1
**Gapdh**	NM_008084.1
**Gusb**	NM_010368.1
**Hprt**	NM_013556.2
**Pgk1**	NM_008828.2
**Tubb5**	NM_011655.4

Supplementary Table 4: Number of events (all-cause mortality) and incidence rate/100-person years in the complete CAD cohort. Dichotomous variables were compared using cross-tabulations with chi-square tests (Two-sided p-values).

	**Number of events** (ACKR3 1st tertile vs. ACKR3 2nd/ 3rd tertile)	**IR/100 PY**	**p-value**
**All-cause mortality**	18/18	5.7/2.6	**0.012**

Supplementary Table 5: Multivariable cox-regression analysis with all-cause mortality as independent variable and clinical factors as covariates (Two-sided p-values, no adjustment for multiple comparisons).

Variable	HR (All-cause mortality) (95% CI)	p-value
Age	1.10 (1.05-1.15)	**<0.001**
LVEF%	0.95 (0.92-0.98)	**0.001**
Hyperlipidemia	2.64 (1.00-6.49)	0.051
ASA	0.95 (0.43-2.10)	0.896
Clopidogrel	0.89 (0.36-2.20)	0.896
ACE inhibitors	0.99 (0.40-2.45)	0.982
ARBs	0.71 (0.25-2.07)	0.531
Beta blockers	1.50 (0.56-4.06)	0.422
Statins	0.90 (0.37-2.16)	0.810
ACKR3 1st vs. 2nd and 3rd tertile	0.47 (0.28-0.76)	**0.002**

Supplementary Table 6:

Multivariable cox-regression analysis with all-cause mortality as independent variable and clinical factors as covariates (Two-sided p-values, no adjustment for multiple comparisons).

Variable	HR (All-cause mortality) (95% CI)	p-value
**Age**	1.10 (1.05-1.15)	**<0.001**
**Gender**	2.09 (0.93-4.69)	0.073
**LVEF%**	0.95 (0.92-0.98)	**<0.001**
**Hyperlipidemia**	2.46 (0.94-6.42)	0.067
**Diabetes mellitus**	2.13 (1.03-4.39)	**0.041**
**Smoking**	1.60 (0.67-3.80)	0.287
**ASA**	0.90 (0.39-2.07)	0.805
**Clopidogrel**	0.88 (0.34-2.27)	0.791
**ACE inhibitors**	0.79 (0.29-2.08)	0.627
**ARBs**	0.74 (0.23-2.37)	0.614
**Beta blockers**	1.43 (0.51-4.02)	0.503
**Statins**	1.12 (0.46-2.77)	0.801
**ACKR3 1st vs 2nd and 3rd tertile**	0.31 (0.14-0.69)	**0.004**

Supplementary Table 7:

Number of events and incidence rate/100-person years in the complete CAD cohort Dichotomous variables were compared using cross-tabulations with chi-square tests (Two-sided p-values).

Event	No. of events (1st tertile vs 2nd/3rd tertile)	IR/100py	p
**All-cause mortality**	19/17	5.8/2.6	**0.008**
**Myocardial infarction**	21/25	6.4/3.8	0.052
**Ischemic stroke**	1/6	0.3/0.9	0.284

Supplementary Figure 1

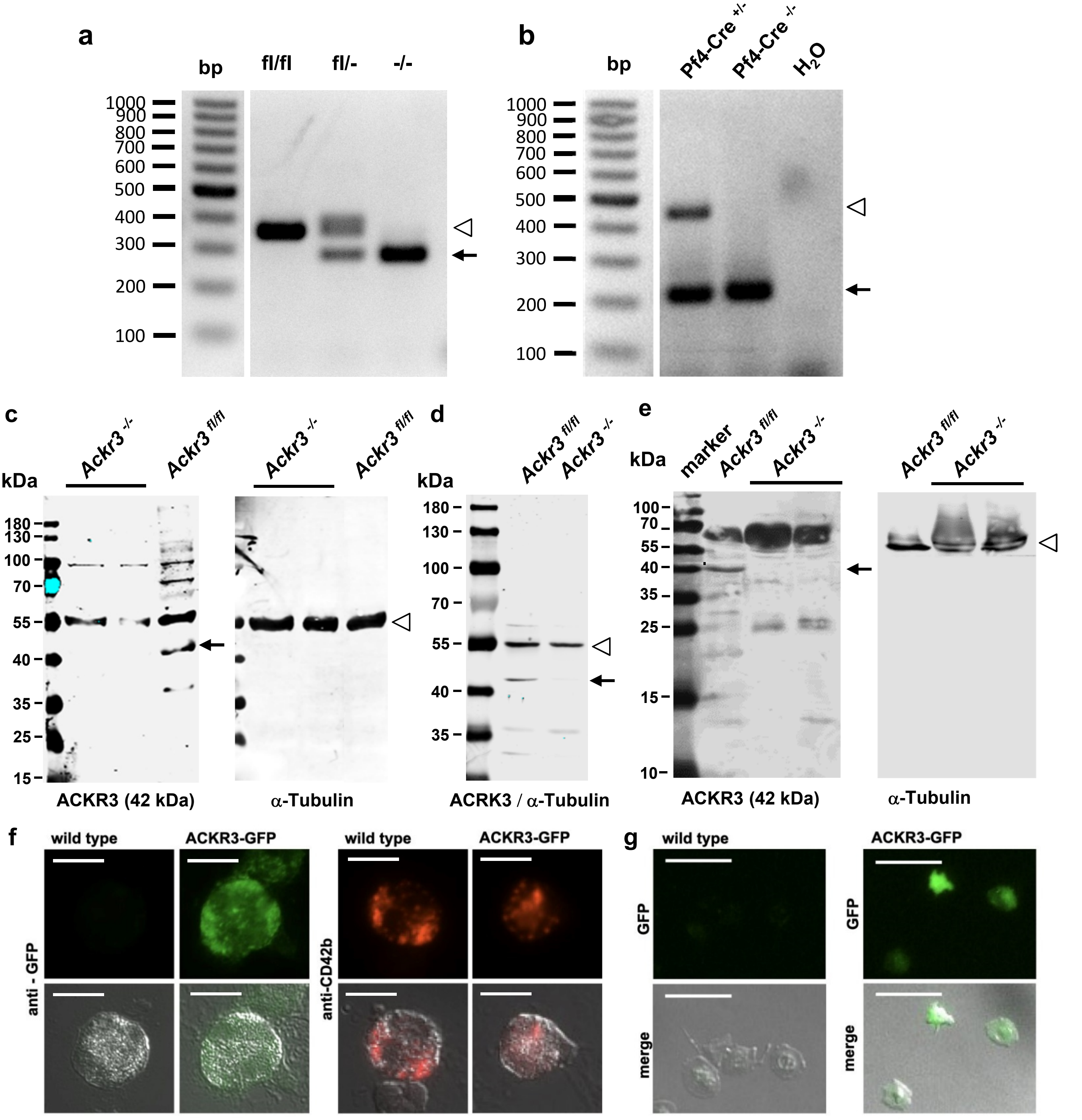

Supplementary Figure 1: Phenotyping of *Ackr3* $^{fl/fl}$ vs *Ackr3* $^{-/-}$ mice. a Representative image of an agarose gel showing the genotyping PCR done with tissues samples from the ear of recently weaned mice for *Ackr3* flox homozygous (fl/fl, 347 bp), heterozygote (fl/-; 347 bp and 277 bp) and wildtype (-/-; 277 bp). **b** Representative image of a 1 % agarose gel depicting the genotyping PCR for Pf4-Cre positive mice done with tissues samples from the ear of recently weaned mice (450 bp; Pf4-Cre $^{+/-}$ and 200 bp PCR control band), Pf4-Cre negative mice (Pf4-Cre$^{-/-}$; 200 bp control band) and a *A. dest* control. **c** 12 % SDS-Page with following immunoblot analysis using the polyclonal Rabbit anti-CXCR7 antibody Abcam 72100. A clear ACRK3 protein band (42 kDa; black arrow) is visible in isolated *Ackr3*$^{fl/fl}$ platelets. This protein band is missing in isolated *Ackr3*$^{-/-}$ platelets. α-Tubulin (55 kDa, white arrow head) expression was used as loading control. **d** 8 % SDS-Page with following immunoblot analysis using the polyclonal Rabbit anti-CXCR7 antibody Novus NBP1-31309. A clear ACRK3 protein band (42 kDa; black arrow) is visible in isolated *Ackr3*$^{fl/fl}$ platelets. This protein band is missing in isolated *Ackr3*$^{-/-}$ platelets. An asterisk (*) marks a residual alpha-Tubulin signal in the ACKR3 staining. a-Tubulin expression (55 kDa, white arrow head) was used as loading control. **e** Immunoblot analysis using the polyclonal Rabbit anti-CXCR7 antibody Abcam 72100. A clear ACRK3 protein band (42 kDa; black arrow) is visible in isolated *Ackr3*$^{fl/fl}$ megakaryocytes. This protein band is clearly missing in isolated *Ackr3*$^{-/-}$ megakaryocytes. a-Tubulin expression (55 kDA, white arrow head) was used as loading control. **f** Representative DIC and fluorescence images of megakaryocytes from a ACKR3-GFP reporter strain with endogen GFP expression and CD42b positive IFF straining. The ACKR3-GFP signal is missing in megakaryocytes extracted from wild type animals. (scale bar: 25 µm) **g** Representative DIC and fluorescence images of platelets isolated from a ACKR3-GFP reporter strain with endogen GFP The ACKR3-GFP signal is absent in platelets isolated from wild type animals. (scale bar: 10 µm)

Supplementary Figure 2

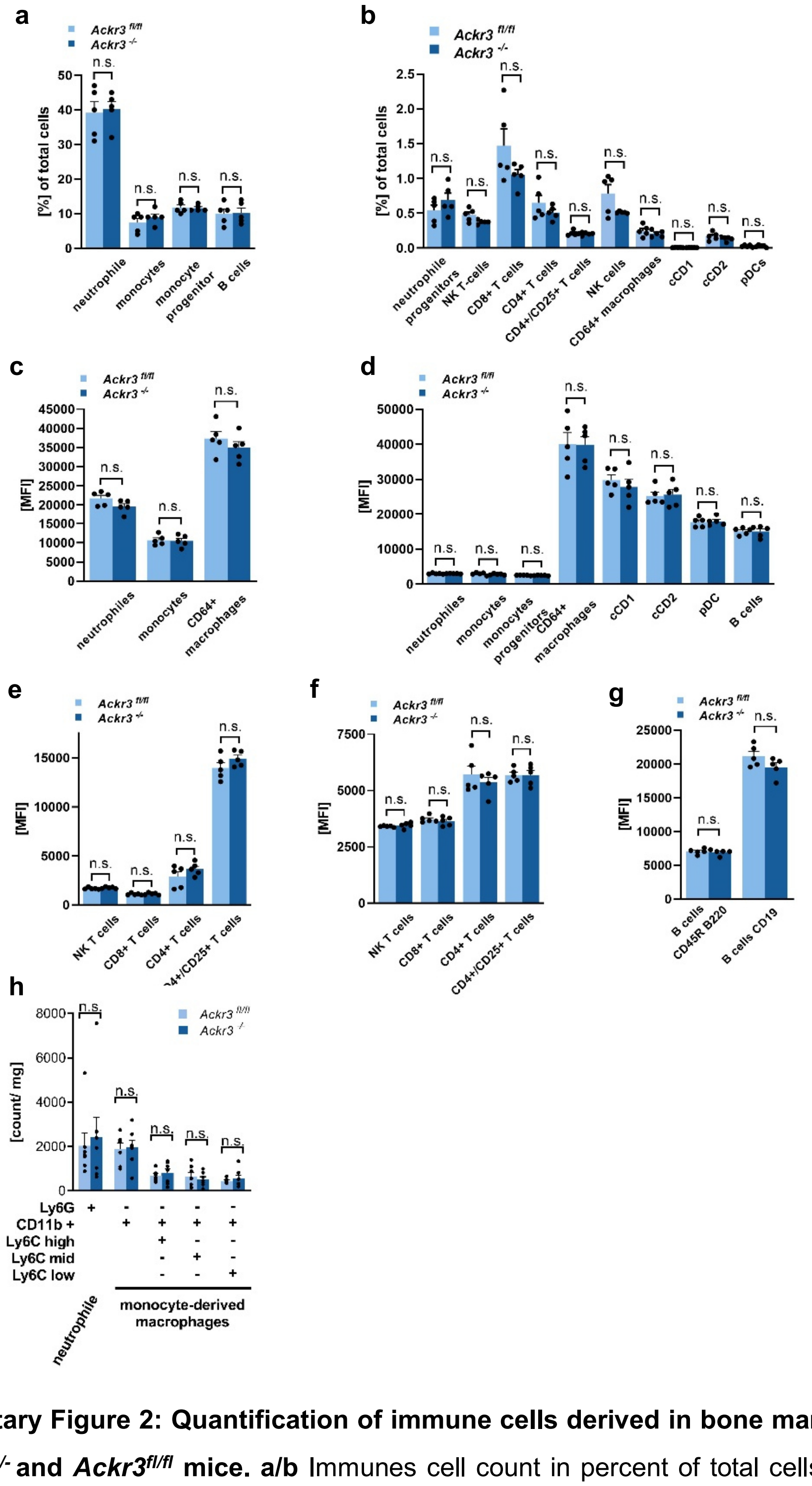

Supplementary Figure 2: Quantification of immune cells derived in bone marrow and spleen from *Ackr3$^{-/-}$* and *Ackr3$^{fl/fl}$* mice. a/b Immunes cell count in percent of total cells within the bone marrow comparing *Ackr3$^{-/-}$* and *Ackr3$^{fl/fl}$* mice. **c** CD11b **d** MHCII expression on immune cells. **e** CD25 expression on immune cells. **f** CD3 expression on immune cells. **g** CD45R/B220 and CD19 expression on B cells. **a-g** n=5; Plotted: Mean±S.E.M.; Statistics: Student's t-test; 95% confidence interval; n.s. = not significant. Exact p-values are given within the source data. **h** Immune cell count per mg spleen tissue in *Ackr3$^{-/-}$* and *Ackr3$^{fl/fl}$* mice. Plotted: Mean±S.E.M.; n=7; Statistics: Student's t-test;, 95% confidence interval; n.s. = not significant. Exact p-values are given within the source data.

Supplementary Figure 3

a

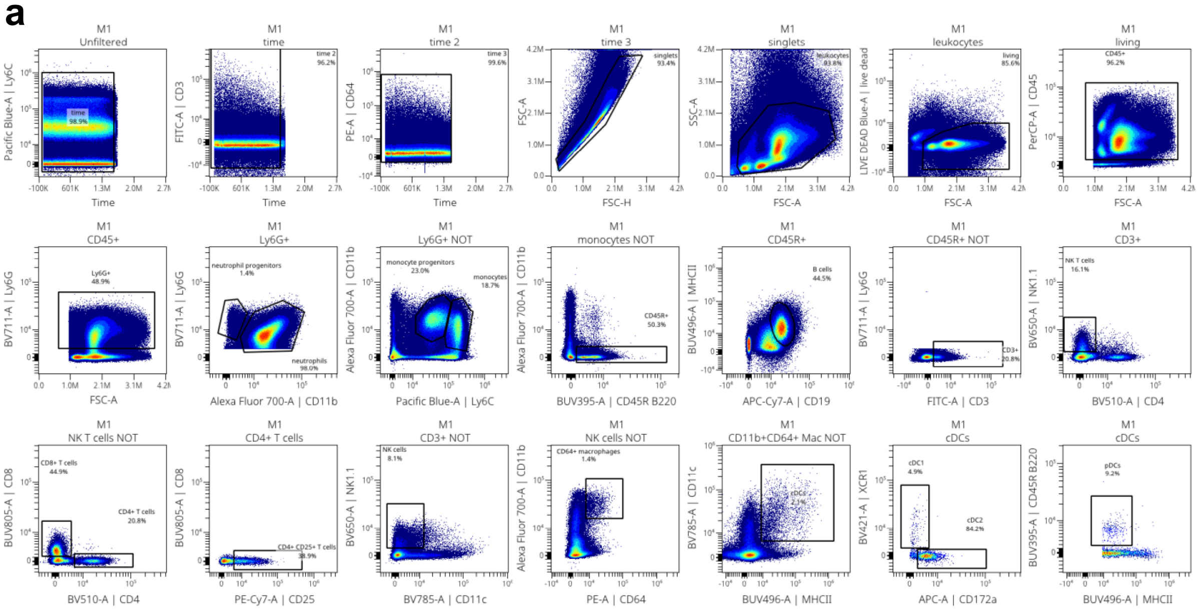

b

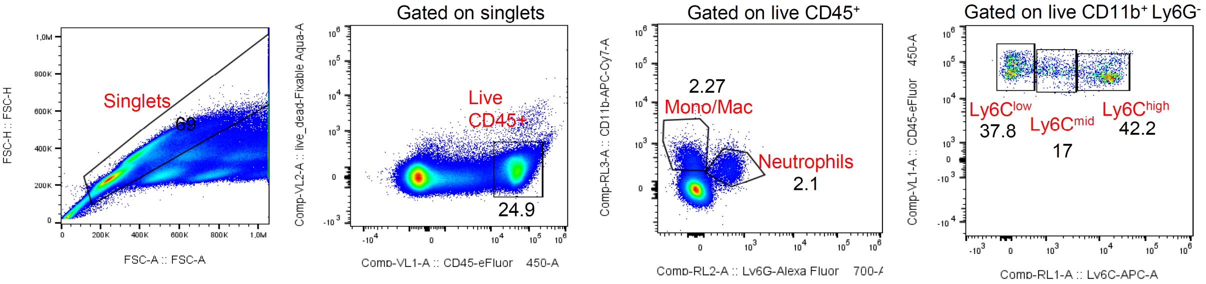

Supplemental Figure 3: Gating strategy for the quantification of immune cells derived in bone marrow and spleen from *Ackr3*$^{-/-}$ and *Ackr3*$^{fl/fl}$ mice. a bone marrow **b** spleen.

Supplementary Figure 4

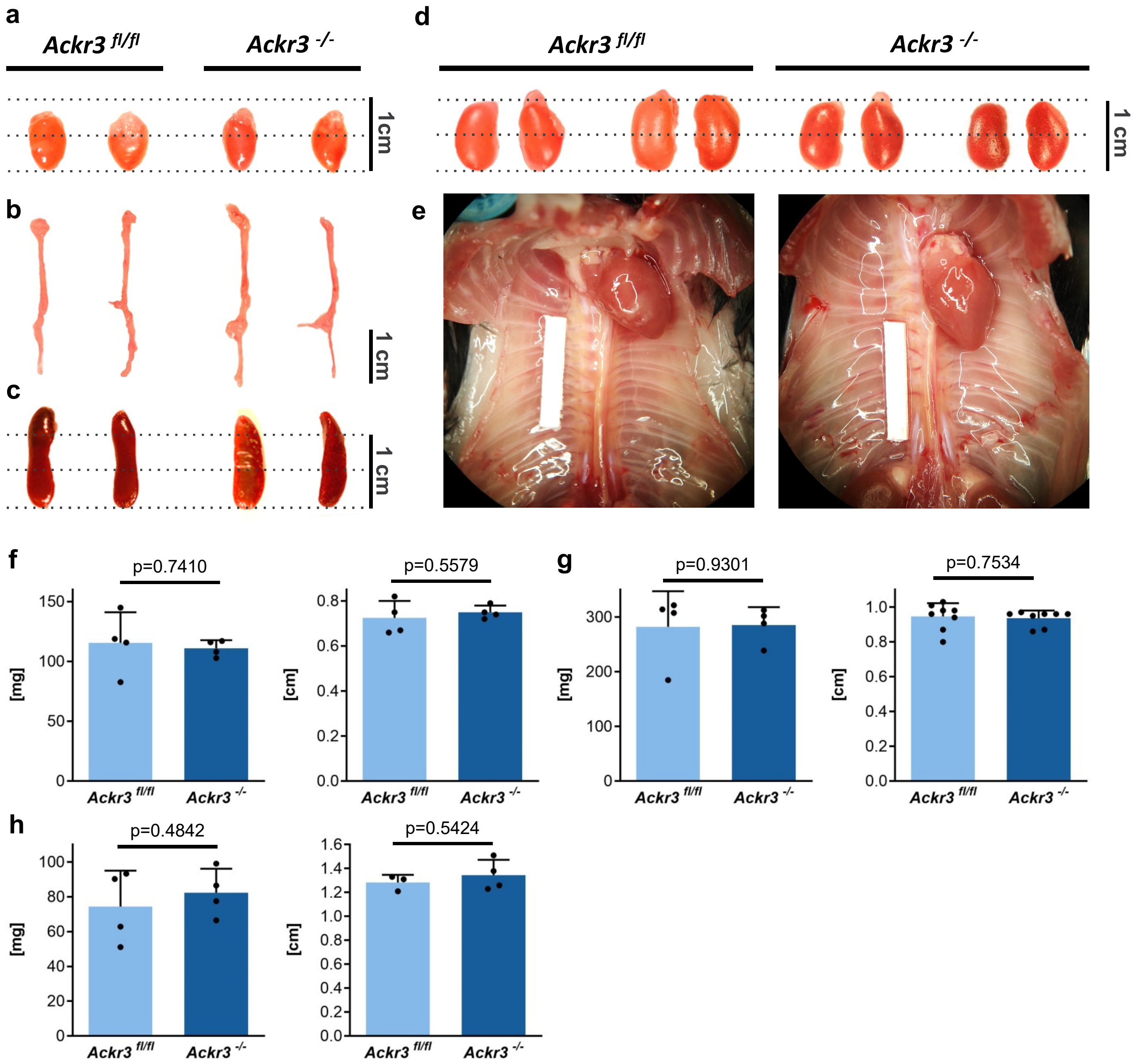

Supplementary Figure 4: Phenotypical comparison of organs from *Ackr3*$^{-/-}$ and *Ackr3*$^{fl/fl}$ mice. a-e representative images **a** heart **b** Aorta **c** Spleen **d** Kidney **e** *In situ* overview of the heart and aorta **f-h** Statistical analysis of organ weight and size **f** heart n=4 **g** kidney n=4/8 **h** spleen n=4. Plotted: Mean±S.D.; Statistics: two-tailed Students t-test, 95% confidence interval.

Supplementary Figure 5

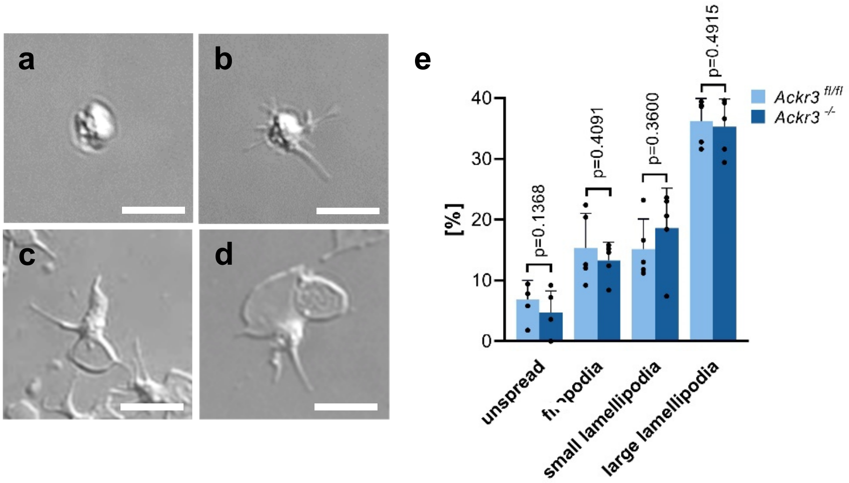

Supplementary Figure 5: Spreading analysis of *Ackr3$^{fl/fl}$* versus *Ackr3$^{-/-}$* animals. a-d Representative images of the various stages of spreading platelets. (scale bar = 5 µm) **a** resting **b** filopodia **c** small lamellipodia **d** large lamellipodia **e** Statistical analysis of the observed spreading stages after 30 min activation with 1 µg/ml CRP. n=5; Plotted: Mean±S.E.M.; Statistics: Student's t-test; 95% confidence interval; ns = not significant.

Supplementary Figure 6

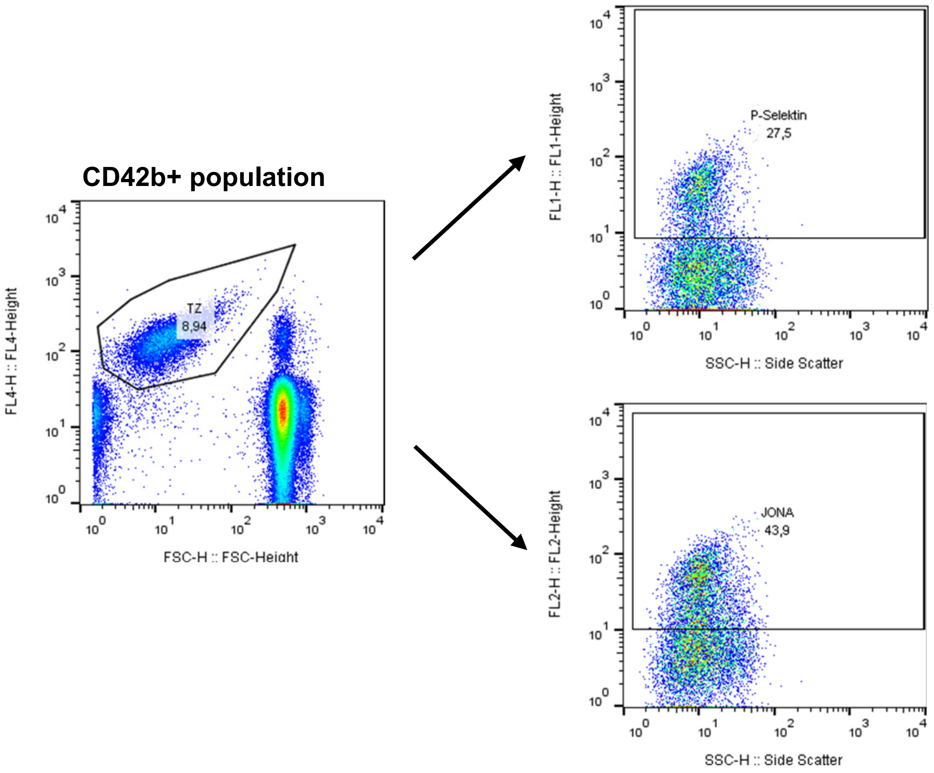

Supplementary Figure 6: Example for our gating strategy for CD42^{+} platelets in whole blood, secondary platelet populations were analyzed for P-Selection or JONA expression.

Supplementary Figure 7

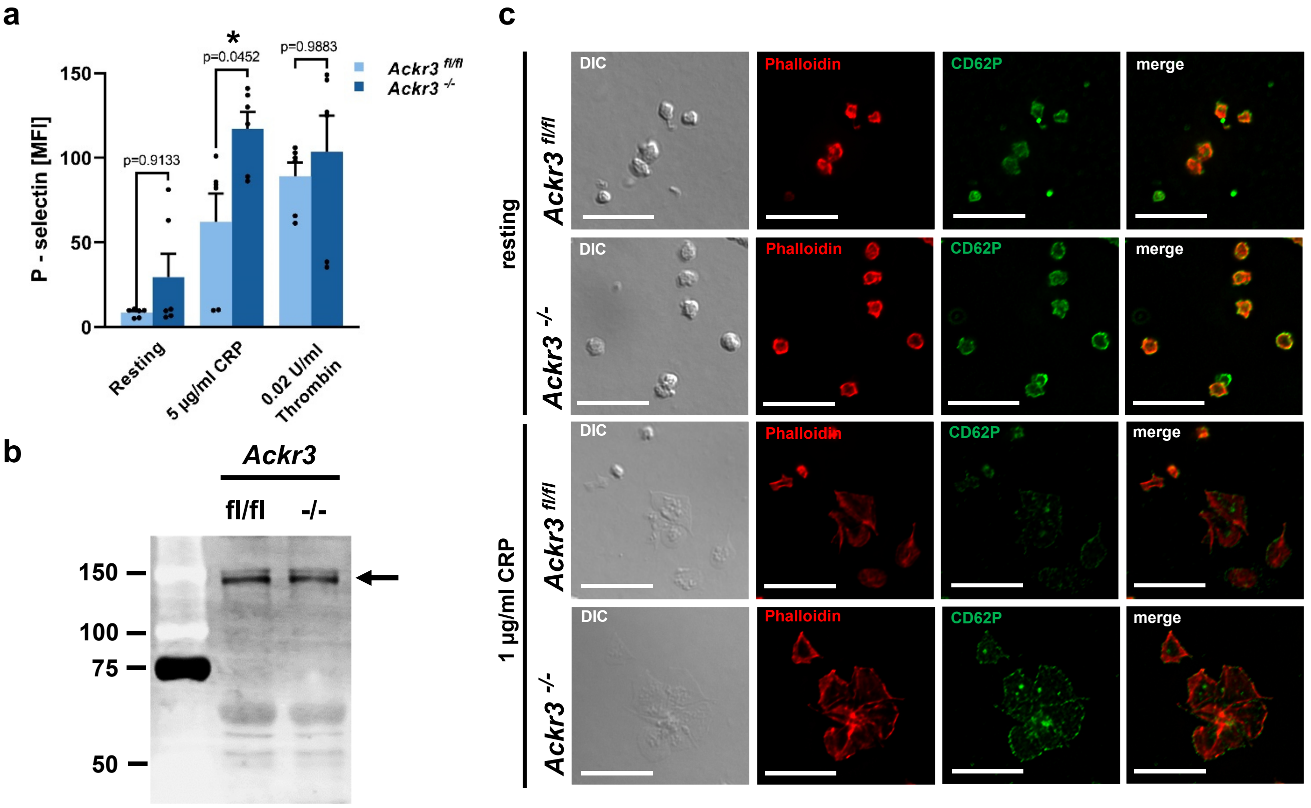

Supplementary Figure 7: P-Selectin expression. a P-Selectin expression upon activation with 5 µg/ml CRP or 0.02 U/ml Thrombin. n=6; Plotted: Mean±S.E.M.; Statistics: one-way ANOVA. **b** Western blot analysis of two sets of platelet lysates generate from resting platelets isolated from *Ackr3*$^{fl/fl}$ and *Ackr3*$^{-/-}$ animals. The specific P-Selectin signal at approx.140 kDa is marked with an arrow. **c** P-Selectin immunofluorescence staining of platelets isolated from *Ackr3*$^{fl/fl}$ and *Ackr3*$^{-/-}$. Staining was performed with Phalloidin-Rhodamin and CD62P – ALEXA488 of stimulated and unstimulated platelets (scale = 10 µm).

Supplementary Figure 8

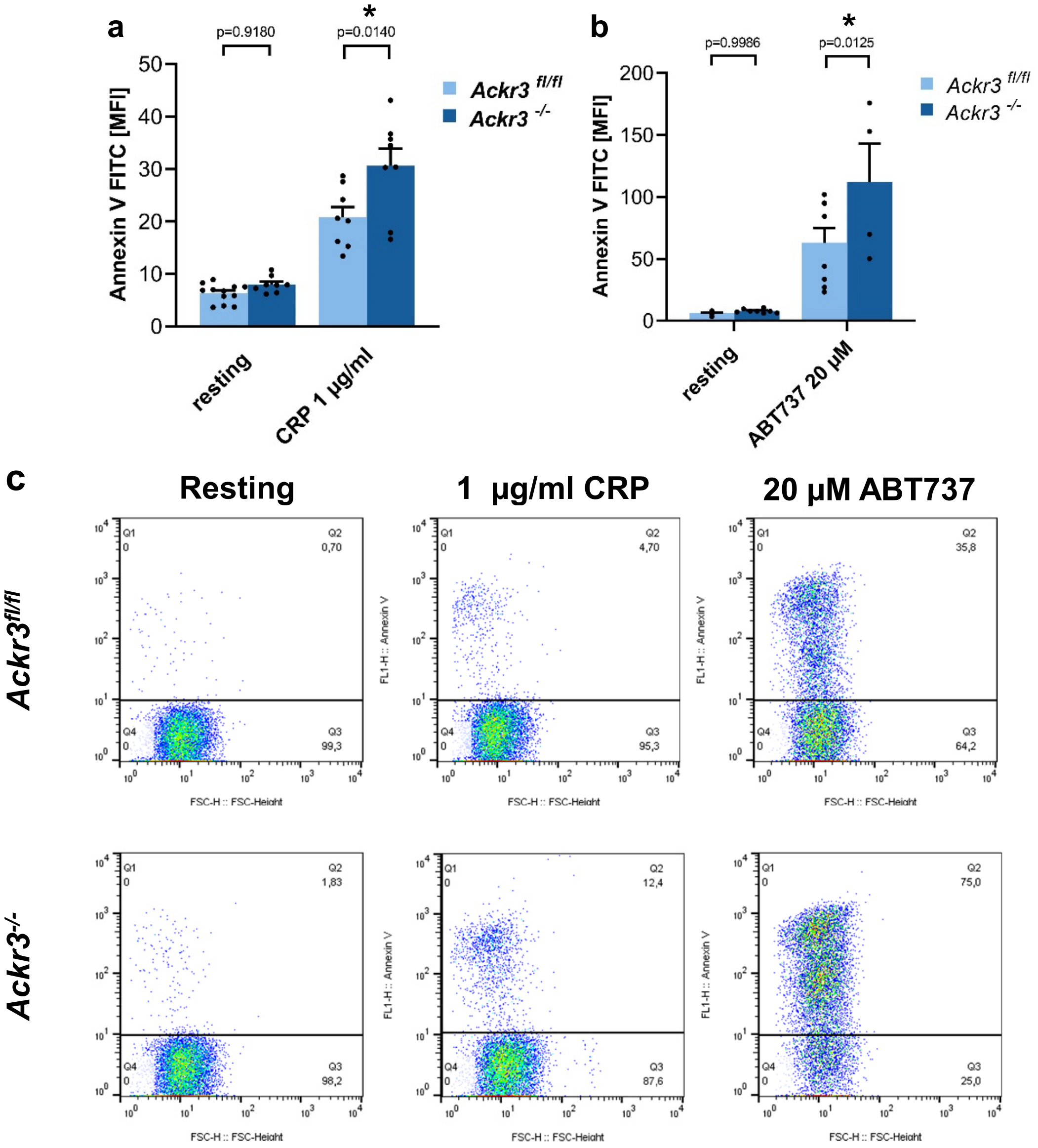

Supplementary Figure 8: Statistical analysis of flow cytometry measurements. a Annexin V expression after stimulation with 1 µg/ml CRP. *Ackr3$^{fl/fl}$* resting: n=12; *Ackr3$^{fl/fl}$* CRP: n=8; *Ackr3$^{-/-}$* resting: n=8; *Ackr3$^{-/-}$* CRP: n=8; Plotted: Mean±S.E.M.; Statistics: two-way Anova. **b** Annexin V expression after treatment with 20 µM ABT737. *Ackr3$^{fl/fl}$* resting: n=12; *Ackr3$^{fl/fl}$* CRP: n=8; *Ackr3$^{-/-}$* resting: n=8; *Ackr3$^{-/-}$* CRP: n=4; Plotted: Mean±S.E.M.; Statistics: two-way Anova. **C** Gating strategy.

Supplement Figure 9

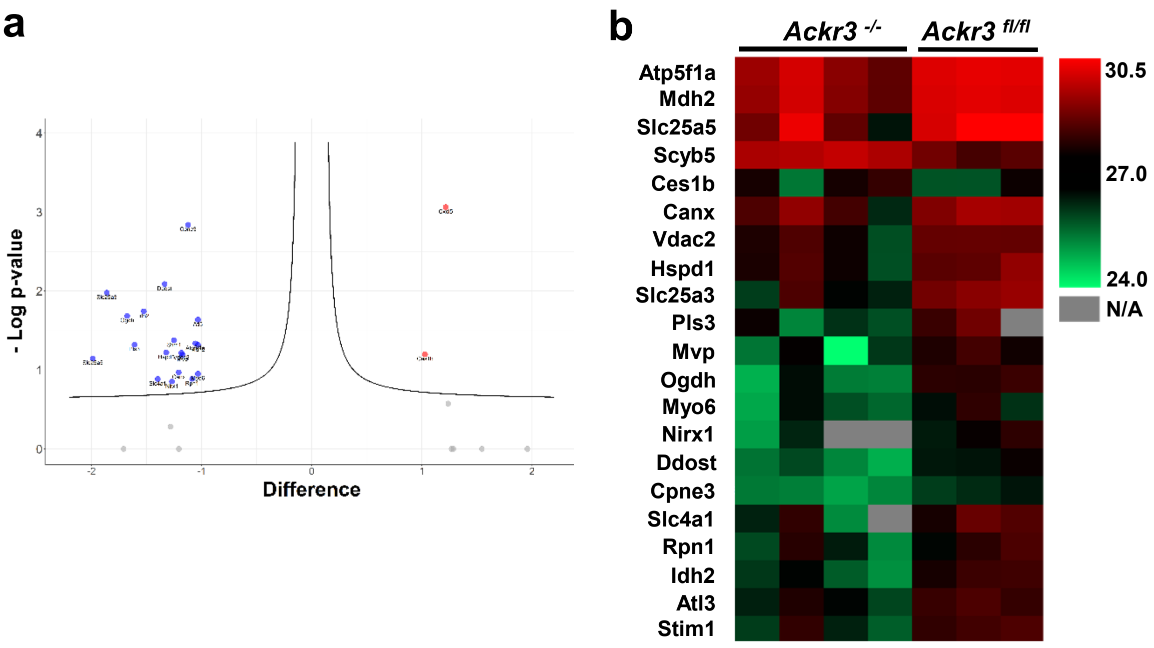

Supplemental Figure 9: Mass spectrometry analysis of activated platelets supernatants (APS). a Volcano plot presentation of the significantly changed proteins in APS. n=4 **b** Heat map presentation of the significantly changed proteins in APS. n=4

Supplementary Figure 10

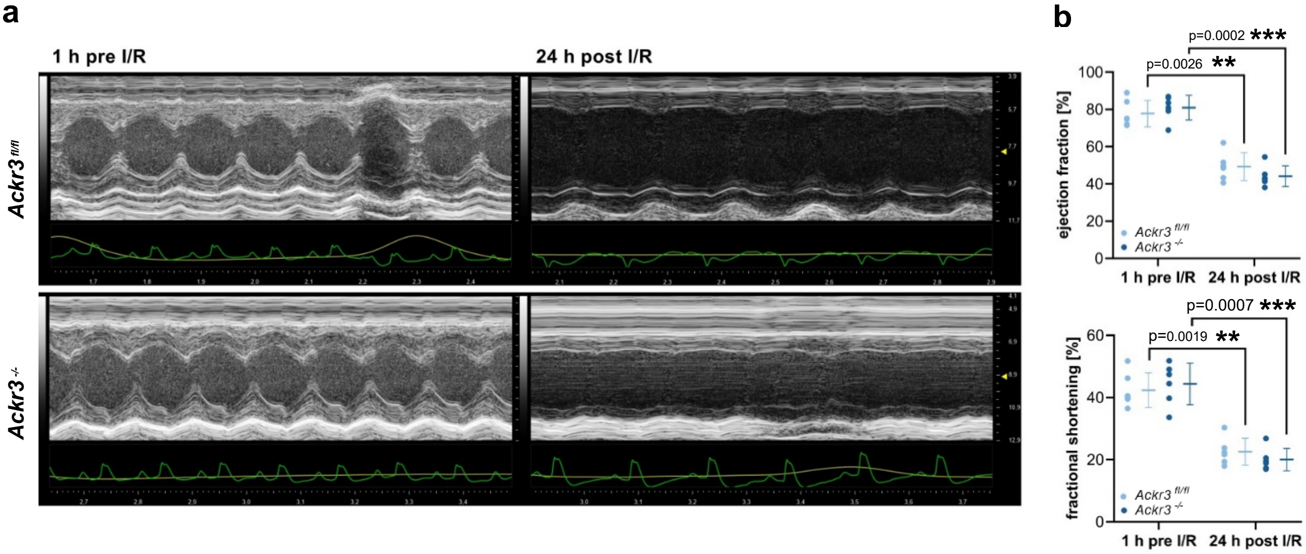

Supplementary Figure 10: a Representative images of ultrasound examinations performed 1 h before and 24 h after ischemia and reperfusion (I/R) in *Ackr3*$^{-/-}$ and *Ackr3*$^{fl/fl}$ animals. **b** Statistical comparison of the ejection fraction and fractional shortening of *Ackr3*$^{-/-}$ and *Ackr3*$^{fl/fl}$ animals before and after I/R. No statistical significant differences between the groups were visible. n=6; Plotted: Mean±S.D.; Statistics: Student's t-test; 95% Confidence interval.

Supplementary Figure 11

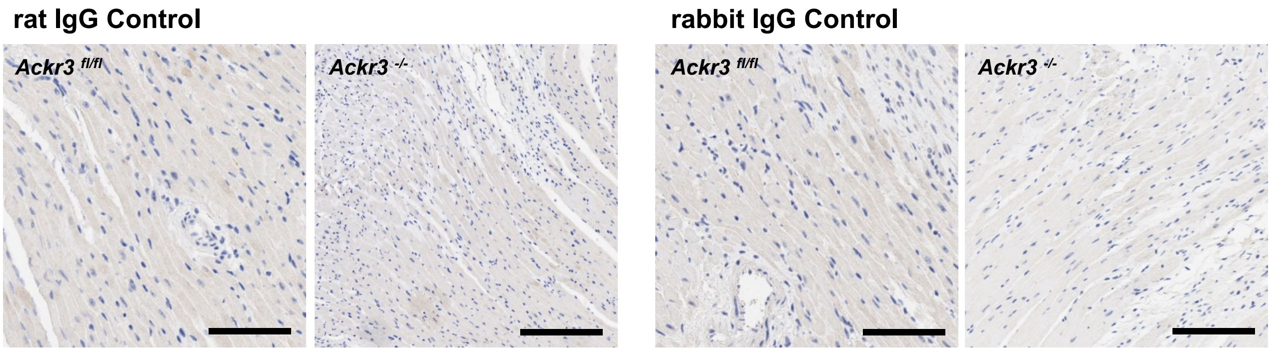

Supplementary Figure 11: IgG Control for the antibodies used in the DAB staining in figure 5 of the manuscript. Scale bar = 50 µm

Supplementary Figure 12

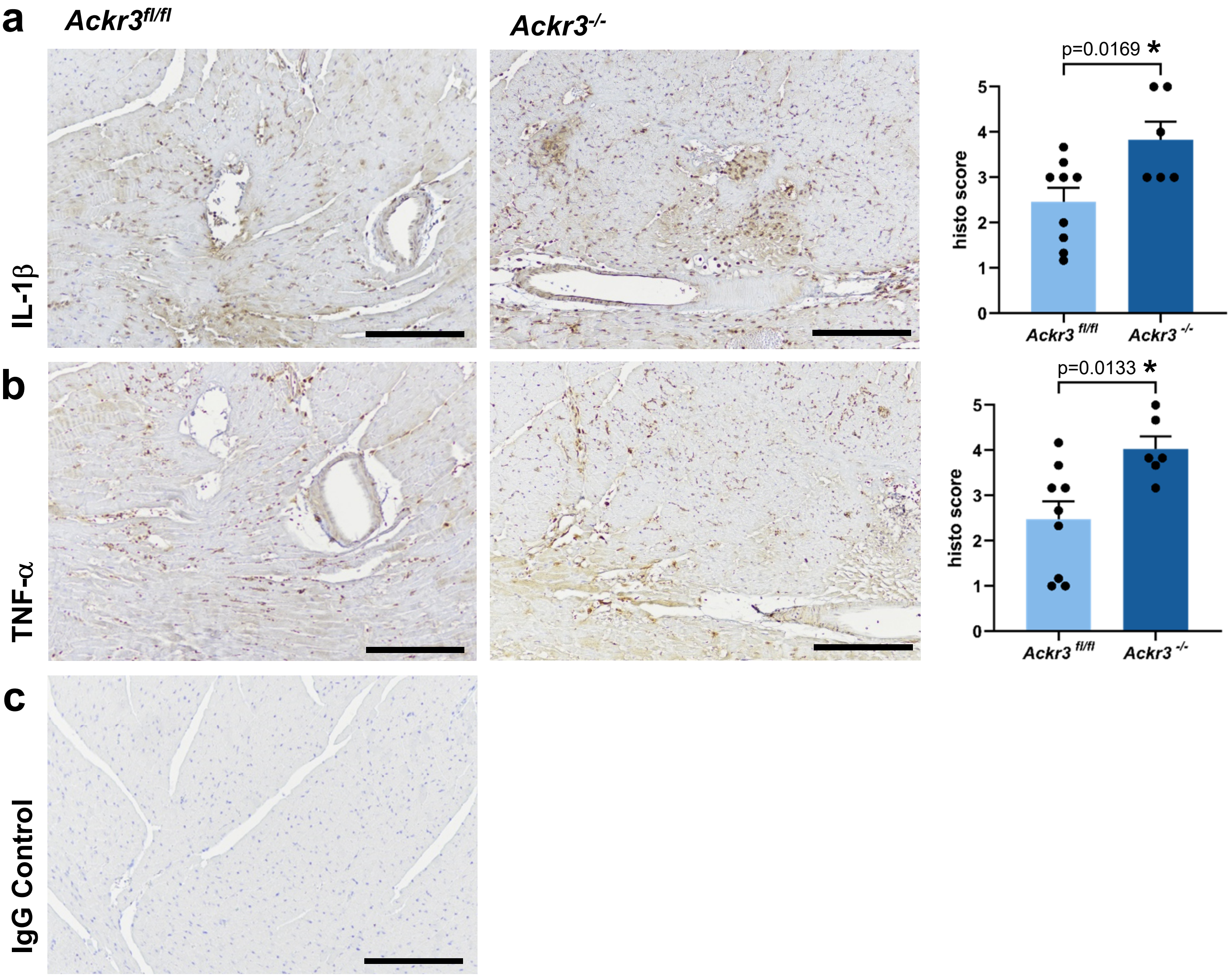

Supplementary Figure 12: DAB immunostaining of the infarct area of heart samples from *Ackr3*$^{-/-}$ to *Ackr3*$^{fl/fl}$ mice 24 h post MI. a Representative image of IL-1beta staining and statistical analysis of the signal. *Ackr3*$^{fl/fl}$: n=9; *Ackr3*$^{-/-}$: n=6; Plotted: Mean±S.E.M.; Statistics: two tailed Student's t-test; 95% confidence interval. Scale bar: 200 µm. **b** Representative images of TNF alpha staining and statistical analysis of the signal. *Ackr3*$^{fl/fl}$: n=9; *Ackr3*$^{-/-}$: n=6; Plotted: Mean±S.E.M.; Statistics: two tailed Student's t-test; 95% confidence interval. Scale bar: 200 µm. **c** Representative image of an appropriate IgG Control. Scale bar: 200 µm

Supplementary Figure 13

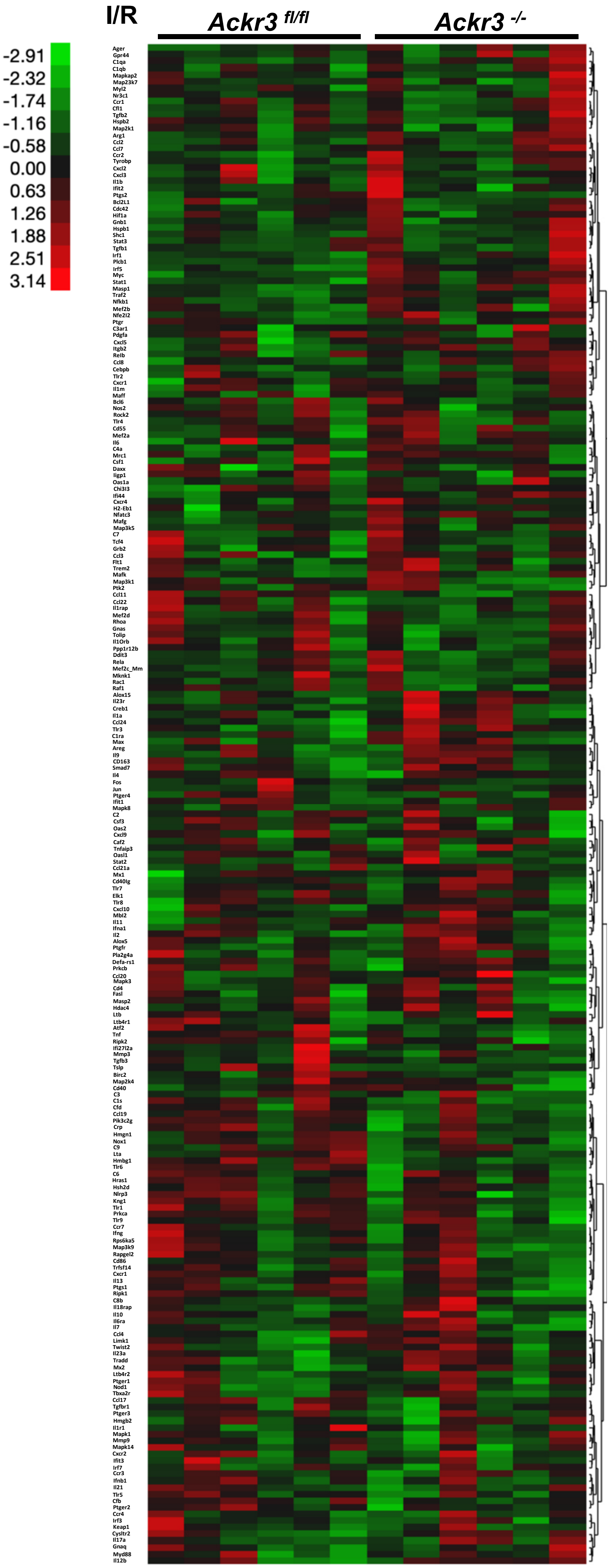

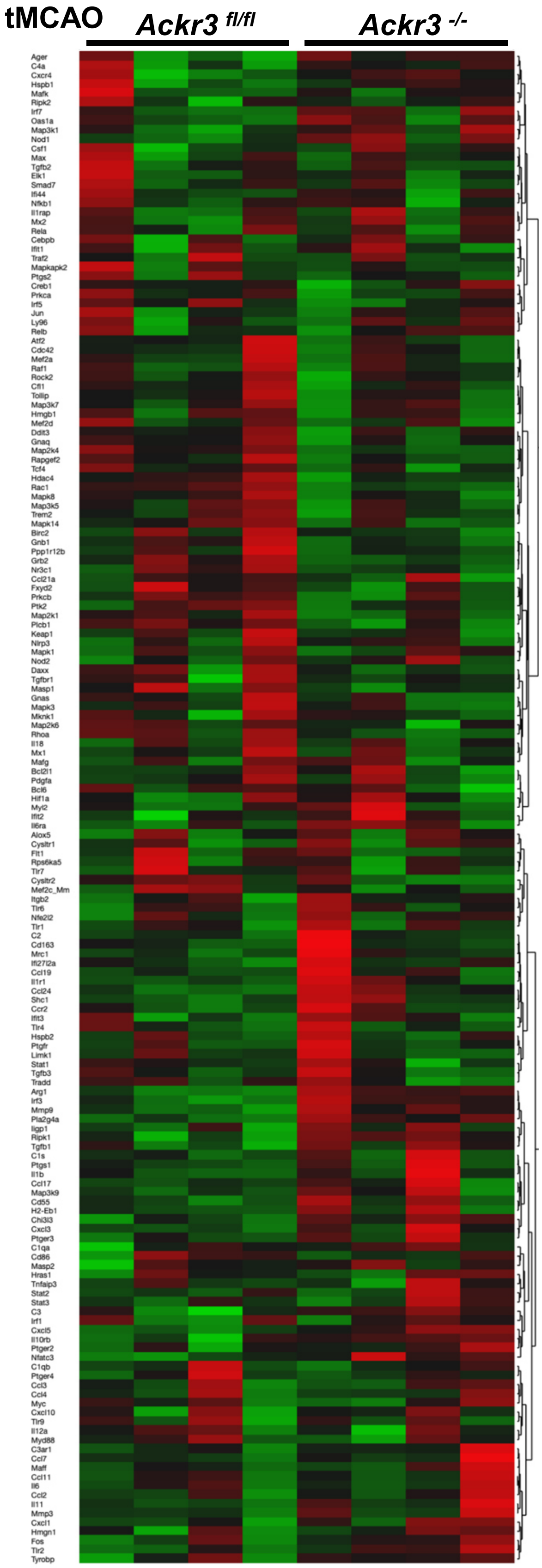

Supplementary Figure 13*:* Nano-String mRNA profiling. Heat map analysis all tested mRNA probes of *Ackr3*$^{-/-}$ and *Ackr3*$^{fl/fl}$ animals after ischemia and reperfusion (I/R) orTransient middle cerebral artery occlusion (tMCAO).

Supplementary Figure 14

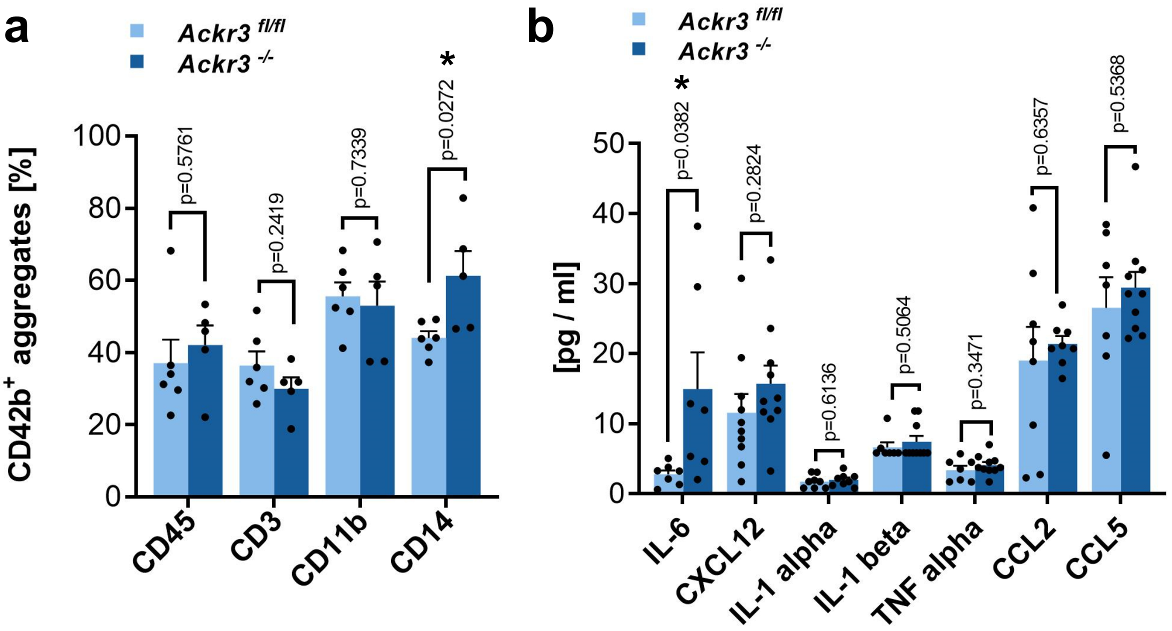

Supplementary Figure 14: Platelet and blood parameter after I/R. a Platelet aggregates in whole blood 24 h post I/R. *Ackr3$^{fl/fl}$* : n=6; *Ackr3$^{-/-}$* : n=5; Plotted: Mean±S.E.M.;Statistics: two tailed Student's t-test; 95 % confidence interval. **b** The chemokine concentrations in the plasma after ischemia and reperfusion on the heart in *Ackr3$^{-/-}$* and *Ackr3 $^{fl/fl}$* animals are altered. A statistical analysis of ELISA data of Plasma derived from *Ackr3$^{-/-}$* and *Ackr3$^{fl/fl}$* animals reveal a significant increase of IL-6 and CXCL5 plasma levels in *Ackr3$^{-/-}$* animals after I/R. Plotted: Mean±S.E.M.;Statistics: two tailed Student's t-test; 95 % confidence interval.

14b) n=	IL-6	CXCL12	IL-1 alpha	IL-1 beta	TNF-alpha	CCL2	CCL5
Ackr3$^{fl/fl}$	7	10	8	7	7	8	7
Ackr3$^{-/-}$	7	10	8	10	10	8	7

Supplementary Figure 15

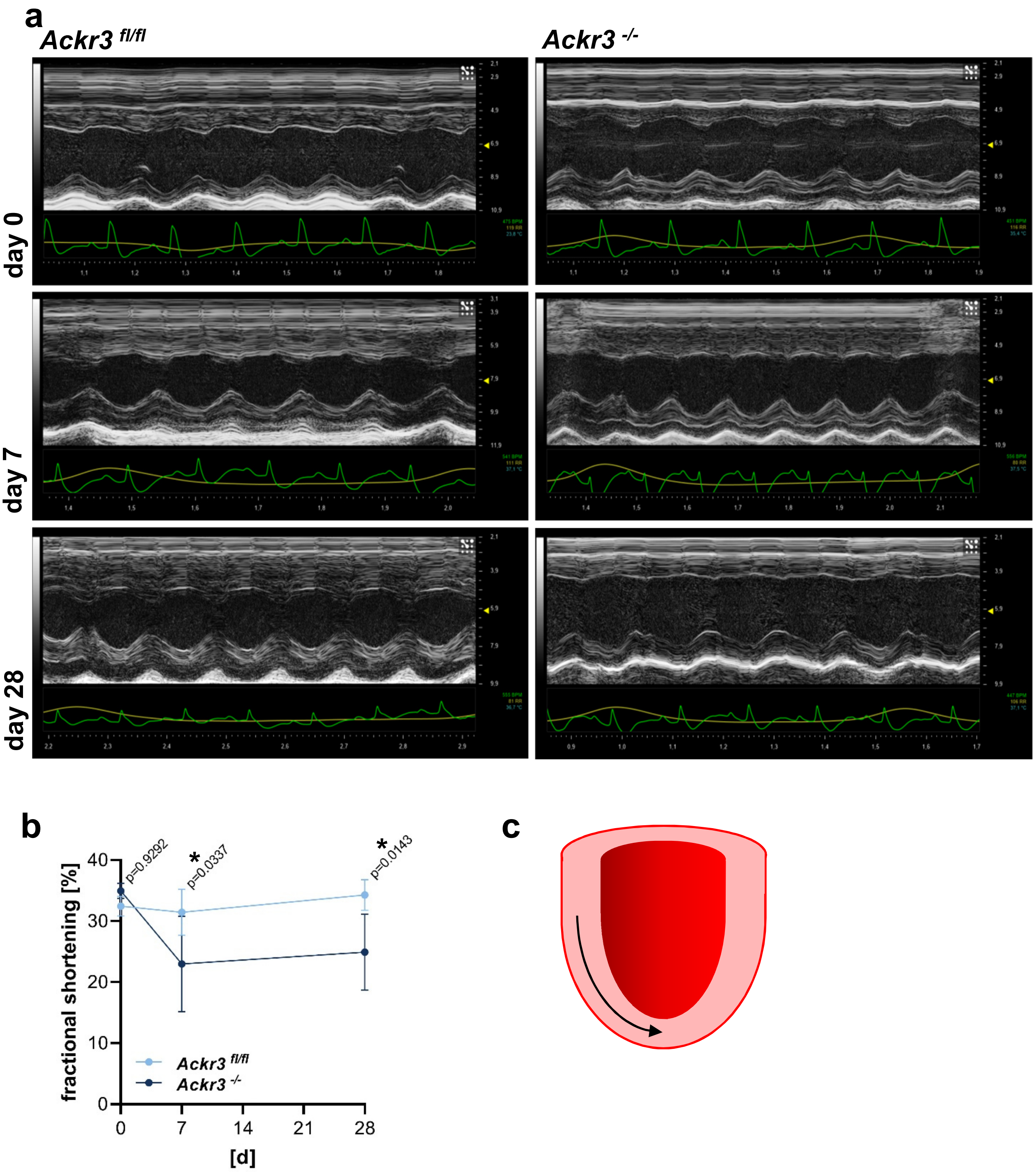

Supplementary Figure 15: Ultrasound analysis pre and 7 days as well as 28 days post myocardial ischemia. a Representative ultrasound images pre I/R and at day 7 and 28 after myocardial ischemia. **b** Statistical analysis of the fractional shorting in after I/R. n=6, Plotted: Mean±S.E.M.; Statistics: one-way Anova. **c** Global longitudinal strain (GLS) graphic.

Supplementary Figure 16

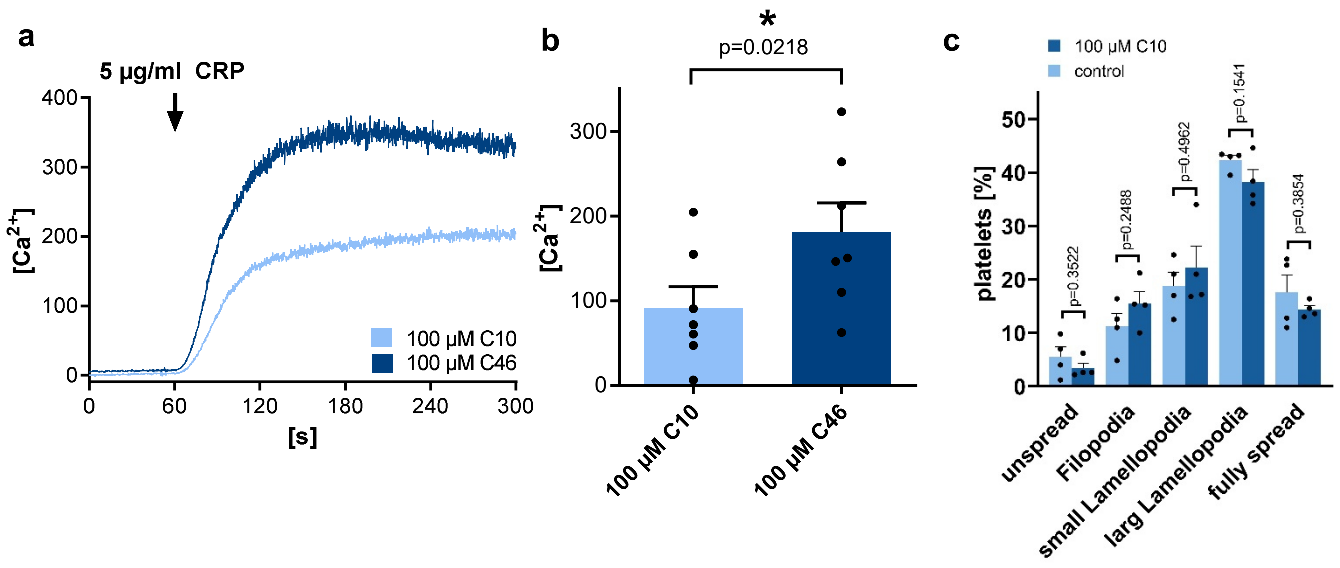

Supplementary Figure 16: Effect of the ACKR3 Agonists on Calcium measurements and platelet spreading. a/b Representative image and statistical analysis of the Calcium increase upon activation with 5 µg/ml CRP in washed platelets treated with 100 µM C10 or C46. n=7; Plotted: Mean±S.E.M.; Statistics: Student's t-test, 95% confidence interval. **c** Spreading of platelets on fibrinogen after 1 µg/ml CRP activation under treatment with 100 µM C10. n=4; Plotted: Mean±S.E.M.; Statistics: Student's t-test, 95% confidence interval.

Supplementary Figure 17

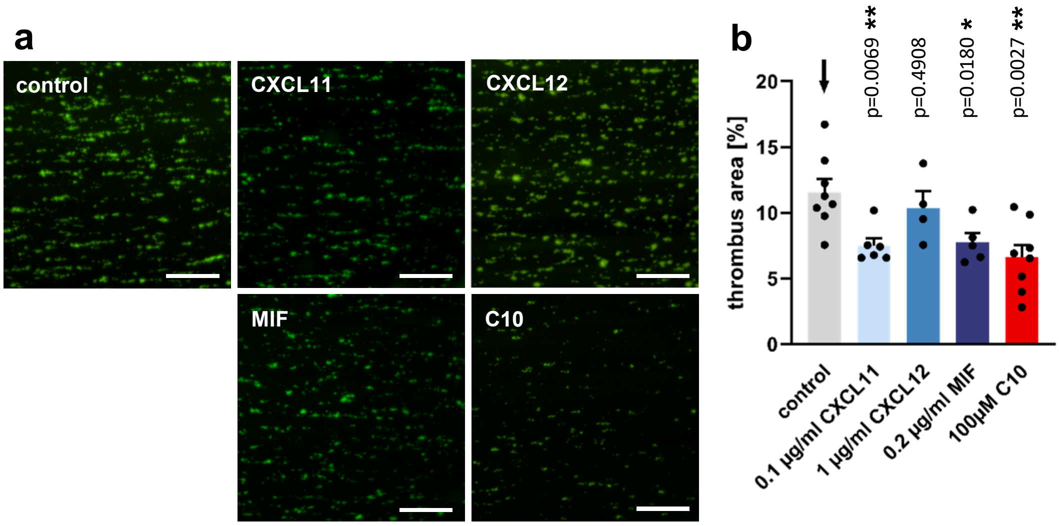

Supplementary Figure 17: Effect of endogen agonists on platelets. a Representative images of flow chamber experiments with endogen agonist. **b** Statistical analysis of flow chamber experiments with endogen agonists. Control n=8; CXCL11 n=6; CXCL12 n=4; MIF n=5; C10 n=8; Plotted: Mean±S.E.M.; Statistics: Student’s t-test; 95% confidence interval.

Supplementary Figure 18

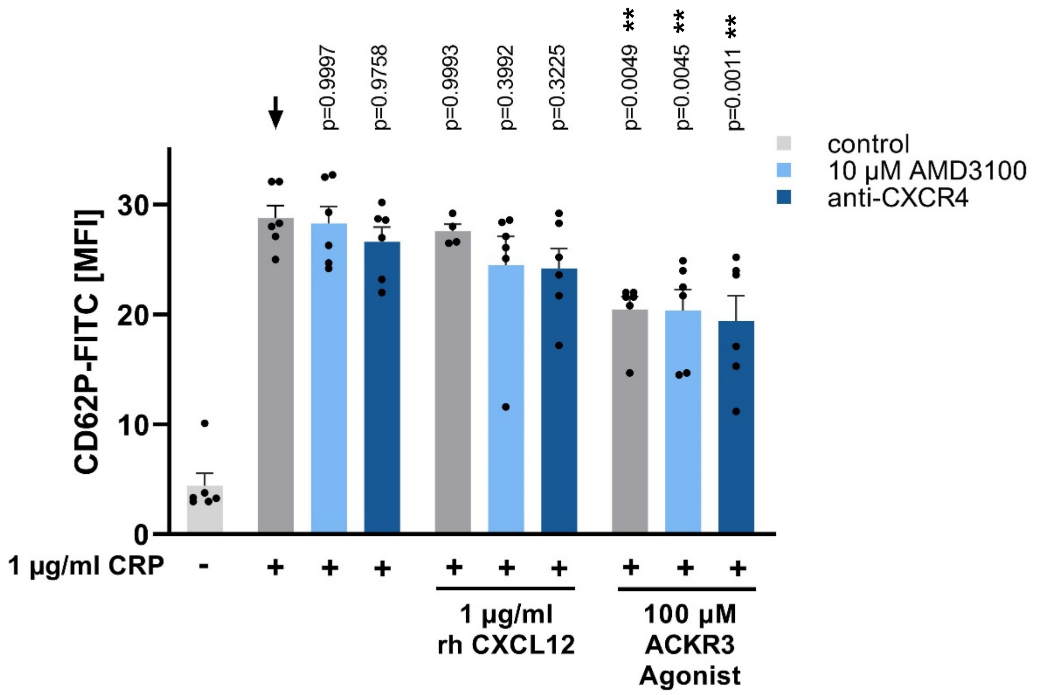

Supplementary Figure 18: Specificity of the ACRK3 Agonist effect. Inhibition of the CXCR4 receptor by AMD3100 or an inhibiting CXCR4 antibody did not alter the significantly reduced CD62P surface expression measured by flow cytometry upon CRP activation after preincubation with the ACKR3 agonist VUF11207. n=6, except CRP + rhCXCL12: n=4, Plotted: Mean±S.E.M.; Statistics: one-way ANOVA.

Supplementary Figure 19

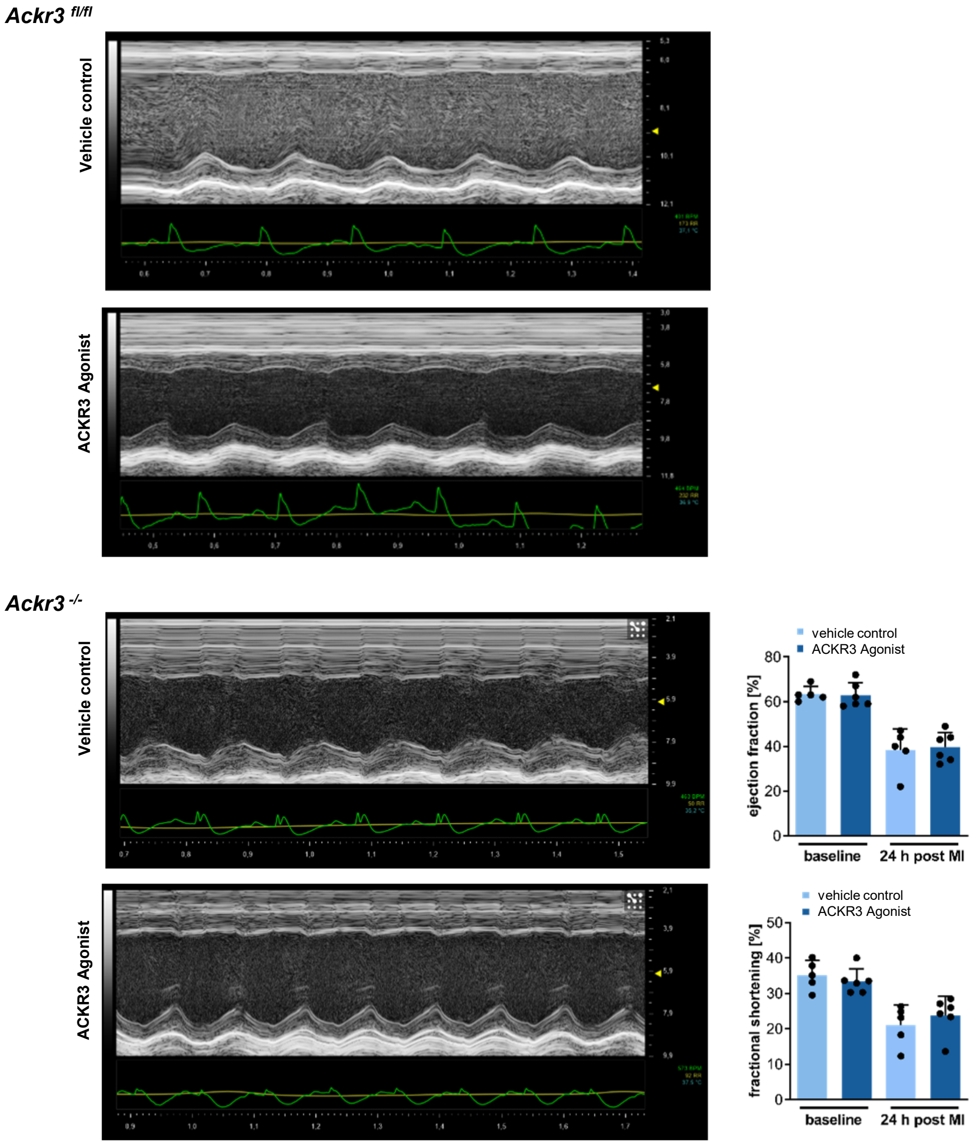

Supplementary Figure 19: Ultrasound analysis pre and 24 hours post myocardial ischemia. Representative ultrasound images pre I/R and at 24 h after myocardial ischemia and statistical analysis of the fractional shorting in after I/R. *Ackr3*$^{fl/fl}$: n=5; *Ackr3*$^{-/-}$: n=6; Plotted: Mean±S.D.
