# Supplementary material for: ACKR3 regulates platelet activation and ischemia-reperfusion tissue injury
Source: Nat Commun. 2022 Apr 5;13:1823. doi: 10.1038/s41467-022-29341-1 (PMC8983782; doi:10.1038/s41467-022-29341-1)
Supplement: Supplementary file 3 — Reporting Summary [file 41467_2022_29341_MOESM3_ESM.pdf]

nature portfolio

Corresponding author(s): Meinrad Gawaz
Last updated by author(s): Nov 3, 2021

# Reporting Summary

Nature Portfolio wishes to improve the reproducibility of the work that we publish. This form provides structure for consistency and transparency in reporting. For further information on Nature Portfolio policies, see our Editorial Policies and the Editorial Policy Checklist.

## Statistics

For all statistical analyses, confirm that the following items are present in the figure legend, table legend, main text, or Methods section.

| n/a | Confirmed | |
|---|---|---|
| ☐ | ☒ | The exact sample size (*n*) for each experimental group/condition, given as a discrete number and unit of measurement |
| ☐ | ☒ | A statement on whether measurements were taken from distinct samples or whether the same sample was measured repeatedly |
| ☐ | ☒ | The statistical test(s) used AND whether they are one- or two-sided<br>*Only common tests should be described solely by name; describe more complex techniques in the Methods section.* |
| ☐ | ☒ | A description of all covariates tested |
| ☐ | ☒ | A description of any assumptions or corrections, such as tests of normality and adjustment for multiple comparisons |
| ☐ | ☒ | A full description of the statistical parameters including central tendency (e.g. means) or other basic estimates (e.g. regression coefficient) AND variation (e.g. standard deviation) or associated estimates of uncertainty (e.g. confidence intervals) |
| ☐ | ☒ | For null hypothesis testing, the test statistic (e.g. *F*, *t*, *r*) with confidence intervals, effect sizes, degrees of freedom and *P* value noted<br>*Give P values as exact values whenever suitable.* |
| ☒ | ☐ | For Bayesian analysis, information on the choice of priors and Markov chain Monte Carlo settings |
| ☒ | ☐ | For hierarchical and complex designs, identification of the appropriate level for tests and full reporting of outcomes |
| ☒ | ☐ | Estimates of effect sizes (e.g. Cohen's *d*, Pearson's *r*), indicating how they were calculated |

*Our web collection on statistics for biologists contains articles on many of the points above.*

## Software and code

Policy information about availability of computer code

| | |
|---|---|
| Data collection | No software was used for data collection. |
| Data analysis | NIS Elements AR 5.21.00 (Build 1481); softwareIBM SPSS Statistics (v26 & v27); GraphPad Prism 9.0.0 |

For manuscripts utilizing custom algorithms or software that are central to the research but not yet described in published literature, software must be made available to editors and reviewers. We strongly encourage code deposition in a community repository (e.g. GitHub). See the Nature Portfolio guidelines for submitting code & software for further information.

## Data

Policy information about availability of data

All manuscripts must include a data availability statement. This statement should provide the following information, where applicable:
- Accession codes, unique identifiers, or web links for publicly available datasets
- A description of any restrictions on data availability
- For clinical datasets or third party data, please ensure that the statement adheres to our policy

The data that support the findings of this study are available as source data. Detailed demographic characterisations of patients are available on reasonable request from the corresponding author.

# Field-specific reporting

Please select the one below that is the best fit for your research. If you are not sure, read the appropriate sections before making your selection.

☒ Life sciences ☐ Behavioural & social sciences ☐ Ecological, evolutionary & environmental sciences

For a reference copy of the document with all sections, see nature.com/documents/nr-reporting-summary-flat.pdf

# Life sciences study design

All studies must disclose on these points even when the disclosure is negative.

| | |
|---|---|
| Sample size | The sample size were calculated and a power of 80 % was assumed. |
| Data exclusions | Data were tested for outliers utilizing the Grubbs test (GraphPad software) were applicable. |
| Replication | At least 3 independent biological replicates were performed, exact n-values are given in the source data and included in the figure legends. |
| Randomization | Random allocation of samples and animals. |
| Blinding | The investigators were blinded to group allocation during data collection for animal experiments and human studies. |

# Reporting for specific materials, systems and methods

We require information from authors about some types of materials, experimental systems and methods used in many studies. Here, indicate whether each material, system or method listed is relevant to your study. If you are not sure if a list item applies to your research, read the appropriate section before selecting a response.

## Materials & experimental systems

| n/a | Involved in the study |
|---|---|
| ☐ | ☒ Antibodies |
| ☒ | ☐ Eukaryotic cell lines |
| ☒ | ☐ Palaeontology and archaeology |
| ☐ | ☒ Animals and other organisms |
| ☐ | ☒ Human research participants |
| ☒ | ☐ Clinical data |
| ☒ | ☐ Dual use research of concern |

## Methods

| n/a | Involved in the study |
|---|---|
| ☒ | ☐ ChIP-seq |
| ☐ | ☒ Flow cytometry |
| ☒ | ☐ MRI-based neuroimaging |

## Antibodies

| | |
|---|---|
| Antibodies used | All antibodies used are listed in the supplemental table 1. |
| Validation | All antibodies used were purchased from well known distributors and were validated before use. Adequate positive and negative controls were performed, such as IgG controls.<br>We routinely titer flow cytometry antibodies in our lab for the best separation between known positive and negative populations. All antibodies used are common and described in the literature. Every antibody was tested from the recommended dilution stated in the data sheet and serially titrated 1:2 for up to 5 times on relevant samples to determine the optimal signal to noise ratio. If no dilution was recommended by the manufacturer we started the titration with 1:20 dilution. |

## Animals and other organisms

Policy information about studies involving animals; ARRIVE guidelines recommended for reporting animal research

| | |
|---|---|
| Laboratory animals | Following mouse strains ( 12 weeks old, female and male animals) were used in the study: C57BL/6J; B6.Cg-Thy1a-(Ackr3)Ackr3tm1Fma-Tg(Pf4-icre)Q3Rsko/J; B6.Cg-Thy1a-(Ackr3)Ackr3tm1Fma-Tg(Pf4-cre)Q3Rsko-Tg(ROSAmT/mG)/J), C57BL/6J-Tg(Pf4-icre)Q3Rsko/J. More information is available in the manuscript and supplemental table 1. |
| Wild animals | No wild animals were used in this study. |
| Field-collected samples | No field collected samples were used in this study. |
| Ethics oversight | The complete statement on ethical approval is provided in the manuscript as given here: "animal handling and all animal experiments were performed according to the German animal protection law and were approved by the local authorities (Regierungspräsidium |

Tübingen M5/17, M20/15, M08/14 and M18/14 - myocardial infarction; University Hospital Essen approval 84-02.04.2017.A106 and Ethics committee of Istanbul Medipol University 16/08/2021-53 - stroke)".

Note that full information on the approval of the study protocol must also be provided in the manuscript.

## Human research participants

Policy information about studies involving human research participants

| | |
|---|---|
| Population characteristics | We included 389 consecutive patients with symptomatic coronary artery disease (CAD; chronic coronary symptom - CCS, n=184; acute coronary symptom - ACS, n=205). Baseline characteristics are given in the supplemental table 2. |
| Recruitment | We included 389 consecutive patients with symptomatic coronary artery disease (CAD; chronic coronary symptom - CCS, n=184; acute coronary symptom - ACS, n=205) (supplemental table 2). ACS was defined as acute chest patient occurring with or without persistent ST-segment elevation as well as positive, or negative in case of unstable angina, cardiac enzymes. Myocardial infarction (MI) was defined as acute myocardial injury with clinical evidence of acute myocardial ischemia and with detection of a rise and/or fall of cardiac troponin (cTn) values with at least one value above the 99th percentile upper reference limit and at least one of the following symptoms of myocardial ischemia: new ischemic changes in electrocardiogram (ECG), development of pathological Q waves in ECG, imaging evidence of new loss of viable myocardium or new regional wall motion abnormality, and identification of a coronary thrombus by angiography. CCS included patients with suspected CAD who presented with stable angina symptoms or newly diagnosed heart failure/left ventricular dysfunction; asymptomatic or symptomatic patients with stabilized CAD <1 year after ACS or patients with recent revascularization, as well as patients >1 year after initial diagnosis or revascularization; and patients with vasospastic or microvascular angina as well as asymptomatic subjects in whom CAD was detected at screening.<br>We consecutively investigated 389 patients with symptomatic CAD. We did not select patients based on any other criteria. The current study has several limitations, however, we do not believe that there exists a significant bias due to patient selection. |
| Ethics oversight | The study was approved by the ethics committee of the Medical Faculty of the Eberhard-Karls-University Tuebingen and the University Hospital Tuebingen (270/2011BO1 and 238/2018BO2) and complies with the declaration of Helsinki and the good clinical practice guidelines. |

Note that full information on the approval of the study protocol must also be provided in the manuscript.

## Flow Cytometry

### Plots

Confirm that:

☒ The axis labels state the marker and fluorochrome used (e.g. CD4-FITC).

☒ The axis scales are clearly visible. Include numbers along axes only for bottom left plot of group (a 'group' is an analysis of identical markers).

☒ All plots are contour plots with outliers or pseudocolor plots.

☒ A numerical value for number of cells or percentage (with statistics) is provided.

### Methodology

| | |
|---|---|
| Sample preparation | Whole blood or isolated platelets were used. Methods are described in details in the methods section of the manuscript. |
| Instrument | FACS-Calibur flow cytometer Becton-Dickinson, Heidelberg, Germany; Attune-NxT machine (Thermo Scientific, Darmstadt, Germany); Aurora spectral flow cytometer (Cytek) |
| Software | FlowJo Software & OMIQ Data Science Platform |
| Cell population abundance | Information is supplied in the methods section of the manuscript. |

Gating strategy

Full gating strategies are included in the methods section and the supplement figures of the manuscript. FSC/SSC settings are the same for murine and human platelets (Voltage FSC: E00 SSC:266). For washed platelets (isolated platelets) and platelet rich plasma (PRP) we only get one population in this gate which are platelets. When using whole blood we stain platelets with a platelet-specific antibody for CD42b and set a gate for the CD42b positive cells.
Gating strategy for multi panel flow cytometry:
Artefacts were excluded using three different time gate settings. Subsequently, doublets were excluded by side- and forward scatter width and height. Then, all populations were gated on forward and side scatter to identify lymphocytes by size and granularity. After these steps live cells were gated based on utilization of viability dye followed by gating on CD45+ cells.
Mouse bone marrow cell populations were then defined as follows: neutrophils as Ly6G+CD11b+ cells; monocytes as Ly6G-Ly6ChiCD11b+ cells, monocyte progenitors as Ly6G-Ly6C+CD11b+ cells B cells as Ly6G-Ly6C-CD11b-CD45R+CD19+MHC II+ cells, CD4 T cells as Ly6G-Ly6C-CD11b-CD45R-CD3+CD4+ cells, CD8 T cells as Ly6G-Ly6C-CD11b-CD45R-CD3+CD8+ cells, NK T cells as Ly6G-Ly6C-CD11b-CD45R-CD3+NK1.1+ cells, NK cells as Ly6G-Ly6C-CD11b-CD45R-CD3-NK1.1+ cells, CD64+ macrophages as Ly6G-Ly6C-CD45R-CD3-NK1.1-CD64+CD11b+ cells, cDC1 as Ly6G-Ly6C-CD45R-CD3-NK1.1-CD11c+MHC II +XCR1+CD172- cells, cDC2 as Ly6G-Ly6C-CD45R-CD3-NK1.1-CD11c+MHC II+XCR1+CD172- cells, pDCs as Ly6G-Ly6C-CD3-NK1.1-CD11c+MHC II+ CD45R+ cells. Exact gating strategy is provided in supplementary material and is called in the manuscript.

☒ Tick this box to confirm that a figure exemplifying the gating strategy is provided in the Supplementary Information.
